# A model-based economic analysis of pre-pandemic influenza vaccination cost-effectiveness

**DOI:** 10.1186/1471-2334-14-266

**Published:** 2014-05-16

**Authors:** Nilimesh Halder, Joel K Kelso, George J Milne

**Affiliations:** 1 School of Computer Science and Software Engineering, The University of Western Australia, 35 Stirling Highway, Crawley, WA 6009, Australia

**Keywords:** Pre-emptive vaccination, Reactive vaccination, Pandemic influenza, Cost effectiveness, Simulation model

## Abstract

**Background:**

A vaccine matched to a newly emerged pandemic influenza virus would require a production time of at least 6 months with current proven techniques, and so could only be used reactively after the peak of the pandemic. A pre-pandemic vaccine, although probably having lower efficacy, could be produced and used pre-emptively. While several previous studies have investigated the cost effectiveness of pre-emptive vaccination strategies, they have not been directly compared to realistic reactive vaccination strategies.

**Methods:**

An individual-based simulation model of ~30,000 people was used to examine a pre-emptive vaccination strategy, assuming vaccination conducted prior to a pandemic using a low-efficacy vaccine. A reactive vaccination strategy, assuming a 6-month delay between pandemic emergence and availability of a high-efficacy vaccine, was also modelled. Social distancing and antiviral interventions were examined in combination with these alternative vaccination strategies. Moderate and severe pandemics were examined, based on estimates of transmissibility and clinical severity of the 1957 and 1918 pandemics respectively, and the cost effectiveness of each strategy was evaluated.

**Results:**

Provided that a pre-pandemic vaccine achieved at least 30% efficacy, pre-emptive vaccination strategies were found to be more cost effective when compared to reactive vaccination strategies. Reactive vaccination coupled with sustained social distancing and antiviral interventions was found to be as effective at saving lives as pre-emptive vaccination coupled with limited duration social distancing and antiviral use, with both strategies saving approximately 420 life-years per 10,000 population for a moderate pandemic with a basic reproduction number of 1.9 and case fatality rate of 0.25%. Reactive vaccination was however more costly due to larger productivity losses incurred by sustained social distancing, costing $8 million per 10,000 population ($19,074/LYS) versus $6.8 million per 10,000 population ($15,897/LYS) for a pre-emptive vaccination strategy. Similar trends were observed for severe pandemics.

**Conclusions:**

Compared to reactive vaccination, pre-emptive strategies would be more effective and more cost effective, conditional on the pre-pandemic vaccine being able to achieve a certain level of coverage and efficacy. Reactive vaccination strategies exist which are as effective at mortality reduction as pre-emptive strategies, though they are less cost effective.

## Background

The threat of an influenza pandemic due to a novel virus strain or subtype to which humans have little or no immunity is a major public health concern. The last four influenza pandemics have ranged in severity from *mild*, having an estimated case fatality ratio (CFR) between 0.01% and 0.08% [1] as in the case of the 2009 pandemic, to *severe*, having an estimated case fatality ratio between 0.74% and 1.8% [2-4] as in the case of the 1918 pandemic. In recent years, avian influenza subtypes such as H5N1 and H7N9 have begun circulating in domestic bird populations in South-East Asia and China. These subtypes result in high case fatality rates in humans who have contracted influenza from infected birds, having an estimated CFR in patients admitted to hospital of between 30% and 70% [5-10]. This may lead to a major public health disaster if such a virus mutates or reassorts into a form transmissible between humans. Recent research studies highlighted the danger of H5N1 mutation into a form readily transmissible between mammal species, namely ferrets [11-13], demonstrating potential transmissibility between humans.

Health authorities of many countries, often working with the World Health Organisation (WHO), have established pandemic mitigation plans that advocate the use of a range of intervention strategies including social distancing, neuraminidase inhibitor antiviral drugs, and vaccination as the major defences against pandemic associated illness and mortality.

Although vaccination is an effective and long-lasting solution to the threat posed by a pandemic influenza strain, the likely scenario, born out in the 2009 pandemic, is that a newly emerged influenza pandemic will have spread to most parts of the world before a vaccine matched to the pandemic subtype can be produced. A high efficacy influenza vaccine matched to the circulating pandemic strain could take at least 6 months from virus isolation to final vaccine production with current proven techniques [14].

In case of a highly transmissible and pathogenic pandemic, interventions that can be activated without delay will be required to combat pandemic spread until a matched vaccine can be developed. Modelling studies have indicated that rigorously deployed social distancing and antivirals measures could suppress pandemic spread until a vaccine becomes available [15,16]. However the social disruption and economic cost of maintaining these measures for many months could be very large, and may render them practically infeasible.

An alternative (or possibly complementary) intervention measure is a pre-pandemic vaccine: a vaccine produced from isolated influenza virus strains that are considered most likely to emerge as pandemic strains potentially lethal to humans. Such a vaccine could be developed and used in anticipation of a pandemic, and would not be subject to the development delay of a reactively developed vaccine. In recent years, the candidate H5N1 vaccines have been advocated for pre-emptive use to combat against a potentially lethal pandemic that could cause by H5N1 [17-27]. Modelling of pre-emptive vaccination also suggested that pre-pandemic vaccines could mitigate such pandemics [28-31].

Several modelling studies [16,28-35] have examined the cost-effectiveness of the use of vaccination as a pandemic influenza mitigation measure. Milne *et al*. [28] demonstrated the mitigating effect of vaccination with pre-pandemic H5N1 vaccines. Baguelin *et al*., Prosser *et al*., and Lugnér *et al.*[32,33,35] examined the economic outcomes of various influenza vaccination strategies; the studies of Lee *et al*., Newall *et al*., and Khazeni *et al.*[30,31,34] included strategies involving antiviral drugs as well as vaccination; while the studies of Andrasdóttir *et al.* and Sander *et al.*[16,29] also included various social distancing strategies in their analysis. Recently, we have investigated the cost-effectiveness of reactive vaccination taking into account the probable 6-month delay in vaccine availability, with and without combined social distancing and antiviral interventions [15]. The economic outcomes and cost effectiveness analysis of reactive vaccination in conjunction with social distancing and antiviral drugs were also investigated in [16,35].

However, no previous study has explicitly addressed the cost-effectiveness of pre-emptive vaccination strategy in highly plausible scenarios where pre-emptive vaccination is compared to reactive vaccination with a 6-month delay, and where both vaccination strategies are considered with and without rigorous social distancing and antiviral use.

This modelling study addresses this knowledge gap using an established individual-based simulation model of a developed country [15,36-42] by simulating both pre-emptive and reactive vaccination strategies, combined with a range of social distancing and antiviral measures, for plausible future pandemics with different pandemic transmissibility and severity characteristics. The costs to society of each strategy and pandemic scenario stemming from medical treatment, pharmaceutical costs and productivity losses are calculated with an economical model, and the effectiveness of each strategy is assessed in terms of Life Years Saved (LYS). This work continues and completes a sequence of previous studies by the authors on pandemic influenza vaccination: in [28] we assessed the potential effectiveness of pre-pandemic vaccines, but did not address cost-effectiveness or reactive vaccination strategies; while in [15] we assessed the cost-effectiveness of reactive vaccination strategies combined with plausible social distancing and antiviral measures, but did not address pre-pandemic vaccines.

## Methods

In this section we describe the simulation model and economic analysis used in this study, its application to pre-pandemic vaccination. Readers who are familiar with individual-based epidemic spread modelling and cost-effectiveness analysis, and the previous studies of the authors in particular, may be most interested in the “Pre-emptive vaccination” and “Vaccination scenarios” subsections.

### Simulation model

A detailed, individual-based simulation model of a community in Western Australia (Albany, population ~30,000) was used to simulate the dynamics of an influenza pandemic, under various public health mitigation strategies, and for pandemics of different transmissibility and severity characteristics. Data produced by the model indicating the infectious history of each individual in the community was used to determine health outcomes of each individual, considering symptomatic or asymptomatic infection, hospitalisation, ICU treatment, and death. Together with productivity losses due to illness and the interventions themselves, these outcomes were used to estimate the cost and cost effectiveness of alternative intervention strategies.

The *Albany* simulation model used in this study has been used previously in a series of studies addressing the effectiveness and cost effectiveness of various public health pandemic influenza mitigation measures, including the cost effectiveness of reactive vaccination strategies with respect to the delay in vaccine development [15], the economic evaluation and cost effectiveness analysis of social distancing and antiviral drug strategies that were used during the 2009 H1N1 pandemic [39], combinations of antiviral and different social distancing interventions for the future pandemics with mild, moderate and severe characteristics [15,40,41], and the effectiveness of vaccination with pre-pandemic H5N1 vaccines [28]. For the purposes of this study, a model of pre-emptive vaccination was added which allows for different levels of vaccine efficacy and coverage, and includes the ongoing cost of vaccine development and deployment required to keep pre-emptive vaccination coverage current in the population. The model used in the present study carries over all other assumptions from previous studies that do not relate to pre-emptive vaccination.

**Figure 1:**
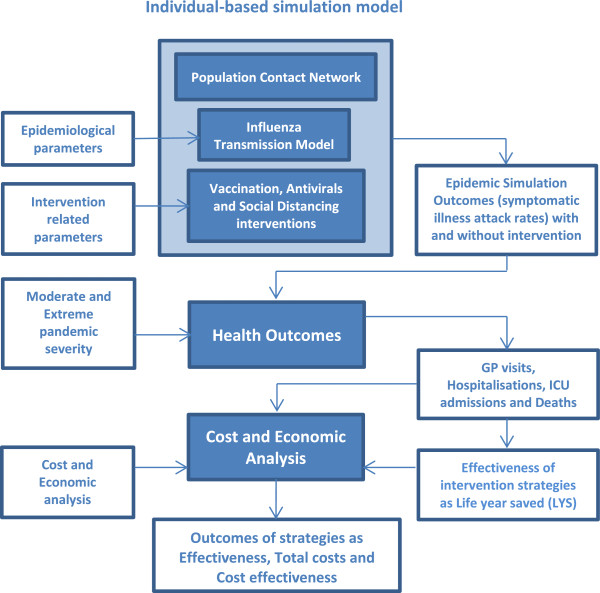
Synopsis of methodology.

A full description of the model appears in Additional file 1; model parameter values are summarised in Table A1.1 of that file. An overview of methodology used for the study is given in Figure 1, while Table 1 describes the terminology used and the definition of pandemic characteristics, together with the alternative vaccination, antiviral and social distancing interventions analysed in the study.

**Table 1:** Terminology and definition

**Terminology**	**Definition**
**Pandemic characteristics**	
**Moderate pandemics**	A moderate pandemic is defined as a pandemic with a transmissibility of R = 1.9 and case fatality rate of 0.25%.
**Severe pandemics**	An extreme pandemic is defined as a pandemic with a transmissibility of R = 2.7 and case fatality rate of 1.5%.
**Vaccinations**	
**Pre-emptive vaccination**	A two-dose pre-emptive vaccination strategy is considered assuming that individuals should be vaccinated with pre-pandemic vaccine annually. This pre-pandemic vaccine should be updated in every 10 years and a pandemic will emerge within a 30 year time frame.
**Reactive vaccination**	A two-dose reactive vaccination strategy is considered assuming that individuals are naïve to the pandemic virus and such reactive vaccines will be available after 6 months of the pandemic emergence.
**Scenarios**	
**Scenario 1**	No pandemic occurs in the 30 year time frame and people have been pre-emptive vaccinated annually.
**Scenario 2**	A pandemic occurs in the 30 year time frame with a virus that does not match pre-pandemic vaccines i.e. vaccine efficacy is 0%. Reactive vaccines will be developed and administrated.
**Scenario 3**	A pandemic occurs in the 30 year time frame with a virus that matches pre-emptive vaccines with vaccine efficacy of 30%.
**Scenario 4**	A pandemic occurs in the 30 year time frame with a virus that matches pre-emptive vaccines with vaccine efficacy of 75%.
**Social distancing and antiviral interventions**	
**Limited duration school closure**	School were closed for 8 weeks of time as follows: for a primary school the whole school was closed if 1 or more cases were detected in the school; in a high school only the class members of the affected class were isolated (sent home and isolated at home) if no more than 2 cases were diagnosed in a single class; however if there were more than 2 cases diagnosed in the entire high school the school was closed.
**Sustained school closure**	All schools were closed simultaneously after a pandemic emerges.
**Community contact reduction**	Community contact reduction was modelled by assuming that on days when the intervention was in effect all individuals made 50% fewer random community contacts. This intervention is also modelled for either limited duration or sustained.
**Antiviral treatment and prophylaxis**	Antiviral drugs used for treatment and prophylaxis of household members (of a symptomatic case) were combined with vaccination and social distancing interventions. It was assumed that 50% of symptomatic individuals would be identified for antiviral treatment and/or prophylaxis, and that treatment and prophylaxis would occur 24 hours after the appearance of symptoms.

### Pandemic characteristics

Two plausible pandemic scenarios (moderate and severe) were defined based on the transmissibility and severity characteristics of past pandemics. The transmissibility of a pandemic was defined in terms of its basic reproduction number R_0_ and associated illness attack rate. The severity of a pandemic was defined in terms of the case fatality rate (CFR).

In this study, a moderate pandemic was defined as having a reproduction number R_0_ of 1.9 and a CFR of 0.25%. The reproduction number of the 1957 and 1968 pandemics has been estimated to be in the range 1.5 and 2.0 [43-45] with CFRs between 0.03% and 0.26% [2,3]. A severe pandemic was defined with a reproduction number R_0_ of 2.7 and a CFR of 1.5%, to reflect the estimated transmissibility and severity characteristics of the 1918 pandemic, thought to have had a reproduction number between 2.0 and 2.9 [44,46-48] with an estimated CFR between 0.74% and 1.8% [2-4].

A proposed pandemic severity index from the US Centers for Disease Control (CDC) uses the CFR for categorising pandemic severity [49]. Severity categories were proposed from category 1, with a CFR < 0.1% to category 5, with a CFR > =2.0%. Our modelled scenarios fit into category 2 for moderate pandemics and category 4 for severe pandemics.

### Pre-emptive vaccination

A two-dose pre-emptive vaccination strategy was considered, as trials of candidate pre-pandemic H5N1 vaccines indicate the need for a two-dose regime to induce immunity [17-27]. It was assumed that pre-emptive vaccination would be an ongoing process and that the pre-pandemic vaccine would be updated and the population re-vaccinated every ten years (with additional vaccination in intervening years at the population birth rate to maintain coverage). This is to ensure that the pre-pandemic vaccine reflects the influenza strains circulating in wildfowl and poultry populations that give most concern from a zoonotic perspective – at the current time these might be H5N1 and H7N9, for example. One possibility for delivery of the pre-pandemic vaccine might be to include it as part of a seasonal influenza vaccination program in each pre-pandemic re-vaccination year, although it may not be possible to achieve high coverage using this strategy. It was assumed that the average time between pandemics was 30 years (based on the spacing of the previous 4 influenza pandemics), so that on average 3 pre-pandemic vaccines would be developed and deployed for each pandemic. Sensitivity analyses to both the 10 year vaccination renewal period and 30 year pandemic return period were conducted.

Full (100%) vaccination coverage was assumed as a baseline value; however since high levels of influenza vaccination coverage may be difficult to achieve without the immanent threat of a highly pathogenic pandemic, sensitivity analyses of different levels of vaccination coverage were conducted. We make no assumption on the rate of pre-emptive vaccination, other than that a certain level of coverage (100% for baseline scenarios, 10%-100% for sensitivity analyses) is achieved before the next pandemic occurs.

Trials of candidate pre-pandemic vaccines for the H5N1 influenza virus have shown seroconversion rates (defined as having a fourfold neutralizing seroconversion rate) between 60 and 90 per cent [18,20-22]. However, these pre-pandemic vaccines may not be closely matched to an emergent influenza strain or may offer only limited cross-strain protection within the virus subtype, thus an efficacy of 30% is assumed. For completeness, a high efficacy pre-pandemic vaccine which closely matches the virus subtype with 75% efficacy was also considered; a scenario where the pre-pandemic vaccine was mismatched (having 0% efficacy) was also considered, in which case we assumed that a vaccine would be developed reactively with a 6-month delay (see below). For individuals aged 65 years or older, we assume that vaccine efficacy will be 80% of the value for those younger than 65. Thus for our assumed pre-pandemic vaccine, we assume a VE of 24% for those 65 years and older, and a VE of 60% for a well-matched vaccine. A review of studies examining antibody responses to influenza vaccination in older individuals found that seroconversion rates were from 60% to 80% those of younger people, depending on the influenza strain [50]. In the case of a well matched vaccine (which we assume as having VE of 75% for younger people), we assume a VE of 60%, which is consistent with a trial that found a VE of 58% for ages 60 and older for a well-matched seasonal vaccine [51].

### Reactive vaccination

A two-dose reactive vaccination strategy was also considered, assuming that individuals are naïve to a future influenza strain and that a two-dose vaccine is essential to achieve immunity [22,23]. During the H1N1 2009 pandemic the first supplies of a suitable vaccine became available after 5–6 months following the appearance of the new strain of H1N1 influenza. In this study, a 6 months delay from the onset of the pandemic to the initiation of a vaccine campaign is assumed, after which the population is vaccinated at a rate of 1% of the population per day. As for the pre-emptive vaccination strategy, full vaccination coverage was assumed.

Availability of a highly effective vaccine is assumed: trials of candidate vaccines for the H1N1 2009 pandemic influenza showed seroconversion rates of vaccines between 82 and 92 per cent [52]. Recent studies estimated that the vaccine effectiveness of the H1N1 2009 pandemic vaccines was between 72% and 87% [53-55]; vaccines with an efficacy of 75% are therefore assumed (60% in individuals aged 65 years or more).

It was assumed that vaccination was prioritised so that age groups known to have higher transmission rates would be vaccinated first. Previous modelling results have indicated that a transmitters-first vaccination strategy is more effective in reducing both attack and case fatality rates than a vulnerable-first approach when timely and wide vaccination coverage is possible [28,56] (note that the issue of vaccination targeting is addressed further in the Discussion section).

### Vaccination scenarios

The reactive vaccination strategy described above is the baseline against which pre-emptive vaccination strategies were compared. Four plausible pre-emptive vaccination strategies were considered and are defined in Table 1.

In scenario 1 an assumption that no pandemic occurs in the 30 year time-frame is made. Thus, there is no pandemic related mortality (and no life years saved), and the only cost incurred is that of pre-emptive vaccination.

In scenario 2, it is assumed that a pandemic does occur in the 30 year time-frame, and the emergent pandemic virus subtype does not match the able pre-pandemic vaccine with which the population has been vaccinated. No protection would be conferred by the pre-emptive vaccine (i.e. a vaccine efficacy of 0%), and it is assumed that a new reactive vaccine would be developed and used after a 6-month delay. The effectiveness of this strategy in saving lives is thus the same as the reactive vaccination strategy, but the costs are the combined costs of pre-emptive and reactive vaccination.

In scenario 3 it is assumed that the virus subtype matches the pre-pandemic vaccine previously used, such that the vaccine efficacy is 30%.

For completeness, scenario 4 considers a pandemic strain which happens to be particularly well matched to the pre-pandemic vaccine, giving a vaccine efficacy of 75%.

### Social distancing and antiviral drug interventions

For the reactive vaccination strategy and each of the pre-emptive vaccination scenarios, three different combinations of social distancing and antiviral drug-based interventions were considered. The first is vaccination alone (i.e. with no additional social distancing or antiviral usage). The second is a combination of social distancing (school closure and community contact reduction) and antiviral treatment and prophylaxis, which is assumed to operate for 8 weeks prior to and overlapping with the peak of the pandemic. The third is the same combination of social distancing and antiviral usage, except that it is assumed to continue for a sustained period (until the time when a reactive vaccination programme would be complete). Full details of non-vaccination interventions are presented in Additional file 1.

Antiviral drug and school closure interventions (either sustained or limited duration) were initiated when specific threshold numbers of symptomatic individuals were diagnosed in the community, which triggered health authorities to activate the intervention response; detailed analyses of these assumptions were presented in previous modelling studies [37,38,57].

Community contact reduction was modelled by assuming that on days when the intervention was in effect all individuals made 50% fewer random community contacts. It was assumed that 50% of symptomatic individuals would be identified for antiviral treatment, that household members of treated individuals would receive antiviral prophylaxis, and that treatment and prophylaxis would occur 24 hours after the appearance of symptoms.

### Influenza transmission model

In the simulation model, the location and activity of each individual in the community is calculated twice per day – during one of the daily cycles (representing the night, evening and morning) individuals were assumed to be at home and made potentially infective contact with all other member of their household. In the other daily cycle (representing day-time activities) individuals were either located in their household, in a workplace, or in a school classroom, and made contact with a subset of individuals in the same location; random community-based contact also occurred during the day cycle. Full details of the human movement and contact model can be found in Additional file 1 and also in [15,28].

Infectious transmission occurred when an infectious and susceptible individual came into contact during a simulation cycle. Following each contact, a new infection state for the susceptible individual (either to remain susceptible or to become infected) was randomly chosen via a Bernoulli trial [37]. Once infected an individual progressed through a series of infection states according to a fixed timeline.

The probability that a susceptible individual would be become infectious after an infectious contact was calculated according to the following transmission function, which takes into account the disease infectivity of the infectious individual *I*_*i*_ and the susceptibility of susceptible individual *I*_*s*_ at the time of contact.

$$\begin{array}{ll} P_{\text{trans}}\left(I_{i},I_{s}\right)= & \beta \times \text{Inf}\left(I_{i}\right)\times \text{Susc}\left(I_{s}\right)\times \text{AVF}\left(I_{i},I_{s}\right)\\ & \times \text{Vaccine}\left(I_{s}\right) \end{array}$$

Each factor contributing to the transmission probability (underlying transmissibility *β*, time-varying transmissibility *Inf*(*I*_*i*_), age-based susceptibility *Susc*(*I*_*s*_), antiviral effectiveness *AVF*(*I*_*i*_, *I*_*s*_), and vaccine effectiveness *Vaccine*(*I*_*s*_)) is described in detail in [15,28] and Additional file 1. The transmission probability coefficient β, capturing the infectivity of the virus strain, was chosen to give unmitigated epidemics with a specific basic reproduction number: R_0_ = 1.9 and 2.7 have been used in this study to capture transmission characteristics for moderate and severe influenza pandemics respectively.

Epidemics were initiated by introducing one randomly located infected seed individual into the population each day, for the duration of the epidemic. All simulations were repeated 40 times with random numbers controlling the outcome of stochastic events (the locality of seeded infected individuals and the outcome of infective contact events) and the results were averaged. Analysis of this simulation model has shown that the 40-run mean attack rate is highly unlikely (95% confidence) to differ by more than 1.2% of the mean attack rate of a much larger set of experiment repeats. In addition, an estimation of confidence interval (95% confidence) for the cost effectiveness ratio for moderate and severe pandemics is presented in Additional File 1: Figure A1.3). For all results we find that the 95% confidence intervals are much smaller than the reported differences in cost effectiveness ratios for different strategies.

We used C++ programing language to create individual-based simulation model and Haskell functional programming for scripting purposes. The data produced by the simulator were fed into an economical model developed using MS excel to generate figures and tables.

### Health outcomes

In order to calculate costs arising from lost productivity and from hospitalisation of ill individuals, an estimate of individual health outcomes (symptomatic illness, hospitalisation, ICU admission and death) is required for each simulated scenario. The number symptomatic illnesses was calculated from simulation results, which generated age-specific attack rates for a pandemic with a particular transmissibility and intervention scenario, assuming that 32% of infections resulted in asymptomatic illness [58]. Pandemic related mortality was then calculated using the number of simulated symptomatic cases and the (symptomatic) CFR according to the pandemic severity (0.25% for a moderate pandemic and 1.5% for a severe pandemic). As in prior cost effectiveness studies conducted using this simulation model [15,35-37], H1N1 2009 pandemic data from Western Australia were used to provide a relationship between the CFR and numbers requiring hospitalisation and ICU treatment. These data indicated a non-ICU hospitalisation to fatality ratio of 32:1 and an ICU admission to fatality ratio of 3:1 and align with those derived by other studies, for example in [59].

### Vaccination cost and economic analysis

As with the method presented in previous cost effectiveness studies using this simulation model [15,39-41], an economic model was used to translate the health outcome of each individual in the modelled population, as derived by the Albany simulation model and the health outcomes model, into the overall pandemic cost burden. The approach taken determines the total economic cost to society incurred during a future influenza pandemic. Total costs involve both direct healthcare costs (e.g. the cost of medical attention due to a GP visit, or for hospitalisation) and costs due to productivity loss due to illness and social distancing interventions. Pharmaceutical costs (i.e. costs related to antiviral drugs and vaccines) are also estimated. Productivity losses due to death were not considered in the analyses leading to the main results of this study, however alternative analyses which include productivity losses resulting from pandemic deaths were conducted and these additional results are presented in Additional file 1.

The calculation of vaccination costs for reactive vaccination and pre-emptive vaccination differ from each other. Reactive vaccines were those which were developed, produced and deployed once the virus subtype of an emergent pandemic was identified, and costs associated with development, manufacture and administration were a once-off cost. In contrast, pre-pandemic vaccines were assumed to be manufactured and administered as an on-going process, with an annual recurrent cost. It was assumed that the pre-pandemic vaccine would be updated every ten years, to ensure that it would reflect the most likely new emergent strain. For each pre-pandemic vaccine developed, it was assumed that the whole population would be vaccinated in the year of development, and that each year thereafter full coverage would be maintained by vaccinating infants (according to the average Australian birth rate). Conservative alternate assumptions that pre-pandemic vaccines would need to be updated and deployed more often (e.g. every 5 or 7 years) were examined in a sensitivity analysis.

All costs are reported in 2012 US dollars using consumer price index adjustments. 2012 US dollar values are used to make the results readily convertible to a wide range of developed countries. A full description of the economic analysis, including cost data used in establishing the overall cost of each pandemic scenario is given in Additional file 1. All future costs and life years were discounted at 3% annually [60]. The time horizon for this analysis is the period up to and including the next influenza pandemic, which was assumed to be on average 30 years based on the spacing of the previous 4 influenza pandemics.

### Cost effectiveness

The cost effectiveness of a given intervention strategy is presented in terms of cost per Life Years Saved (LYS). The numerator used in this cost effectiveness ratio was derived from the total cost arising from a given intervention being applied to the whole community. The denominator was calculated as the difference between years of life lost for an unmitigated pandemic and a pandemic with the intervention applied. Years of life lost data were derived for each simulation from the ages and life expectancies of the individuals who died as a result of the pandemic.

The cost effectiveness of each intervention is presented as a cost in dollars ($) per LYS per person. This was derived by establishing the total cost for a particular intervention strategy and then dividing it by the population of the Albany model, approximately 30,000 individuals, so allowing the results to be applied to a population of any size.

## Results

### Key findings

Assuming that an influenza pandemic occurs every 30 years on average, and that pre-pandemic vaccines are at least 30% effective, a pre-emptive vaccination strategy is more effective, less costly, and more cost effective in terms of cost per life year saved than a reactive vaccination strategy, for all severities and combinations of interventions simulated in this study.

Conversely, if the pre-pandemic vaccines developed are ineffective (failing to provide protection due to strain mismatch), the policy of pre-emptive vaccination is less cost effective than the reactive vaccination strategy, since we assume that a reactive vaccine will also be developed and deployed under these circumstances.

The use of social distancing (SD) and antiviral (AV) interventions in addition to either reactive or pre-emptive vaccination is always more effective than the vaccination alone. The cost effectiveness of combining additional SD and AV with vaccination depends on the efficacy of pre-pandemic vaccines, the duration of social distancing, and the severity of pandemics.

In the following sections we examine the relative effectiveness, total cost and cost effectiveness of pre-emptive and reactive vaccination for all four pandemic scenarios. Since the actual scenario that will occur in a future pandemic is uncertain, we then examine the sensitivity of the relative cost effectiveness of pre-emptive and reactive vaccination policies to key assumptions.

**Figure 2:**
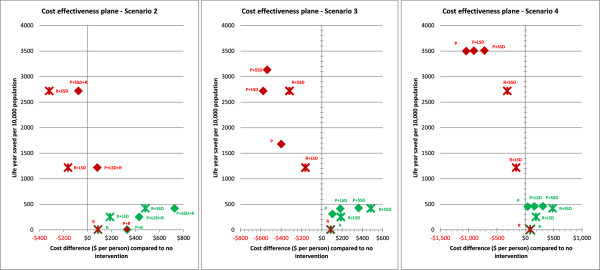
**Cost effectiveness plane for intervention strategies for different scenarios.** Intervention strategies are labelled as R: *Reactive* vaccination, P: *Pre-emptive* vaccination, R + LSD: *Reactive* vaccination + Limited Social Distancing (8 weeks of SC + CCR) + AV, P + LSD: *Pre-emptive* vaccination + Limited Social Distancing + AV, R + SSD: *Reactive* vaccination + Sustained Social Distancing (Sustained SC + CCR) + AV, P + SSD: *Pre-emptive* vaccination + Sustained Social Distancing + AV, SC – School Closure, CCR – Community Contact Reduction, AV – Antiviral treatment for cases and prophylaxis for their household members, LSD – Limited Social Distancing, SSD – Sustained Social Distancing. All LSD and SSD interventions integrate the use of antivirals (AV).

**Figure 3:**
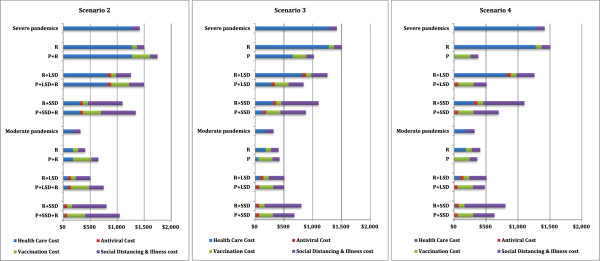
**Cost breakdowns to different costing components.** Interventions strategies are labelled as Figure 2.

The main outcomes of this study, namely the effectiveness (LYS), the total cost, and cost effectiveness as the cost per life year saved ratio of each pre-emptive and reactive vaccination strategy, with and without social distancing and antiviral interventions, for each modelled scenario, and for both moderate and severe pandemics are presented in Tables 2, 3 and 4 respectively. An estimation of confidence interval for the cost effectiveness ratio for moderate and severe pandemics for scenario 2 is presented in Additional File 1. Figure 2 presents a cost effectiveness plane for scenario 2, scenario 3 and scenario 4, which plots each vaccination strategy with and without social distancing and antiviral interventions horizontally according to the costs (compared to no intervention), and vertically according to the effectiveness (life years saved per 10,000 population). Figure 3 presents cost breakdown of different cost components such as health care cost, social distancing and illness cost, antiviral cost and vaccination cost for all simulated pandemic scenarios and interventions.

**Table 2:** Effectiveness (Life years saved per 10,000) of vaccination strategies for moderate pandemics (R = 1.9 and CFR = 0.25%) and severe pandemics (R = 2.7 and CFR = 1.5%)

	**Moderate pandemics (R = 1.9 and CFR = 0.25%)**		
**Scenarios**	**SD and AV intervention**	**Life years saved (LYS) per 10,000 population**	
		**Pre-emptive vaccination**	**Reactive vaccination**
**Scenario 1**: no pandemic	none	-	-
		**Pre-emptive + Reactive vaccination**	**Reactive vaccination**
**Scenario 2**: pandemic with virus which does not match pre-emptive vaccine i.e. vaccine efficacy of 0%	none	4*	4*
	8 weeks SD + AV	253	253
	Sustained SD + AV	421	421
		**Pre-emptive vaccination**	**Reactive vaccination**
**Scenario 3**: pandemic with virus which matches pre-emptive vaccine with efficacy of 30%	none	312	4*
	8 weeks SD + AV	415	253
	Sustained SD + AV	429	421
		**Pre-emptive vaccination**	**Reactive vaccination**
**Scenario 4**: pandemic with virus which matches pre-emptive vaccine with efficacy of 75%	none	453	4*
	8 weeks SD + AV	460	253
	Sustained SD + AV	462	421
	**Extreme pandemics (R = 2.7 and CFR = 1.5%)**		
**Scenarios**	**SD and AV intervention**	**Total cost ($) per 10,000 population**	
		**Pre-emptive vaccination**	**Reactive vaccination**
**Scenario 1**: no pandemic	none	-	-
		**Pre-emptive + Reactive vaccination**	**Reactive vaccination**
**Scenario 2**: pandemic with virus which does not match pre-emptive vaccine i.e. vaccine efficacy of 0%	none	9*	9*
	8 weeks SD + AV	1,219	1,219
	Sustained SD + AV	2,720	2,720
		**Pre-emptive vaccination**	**Reactive vaccination**
**Scenario 3**: pandemic with virus which matches pre-emptive vaccine with efficacy of 30%	none	1,680	9*
	8 weeks SD + AV	2,719	1,219
	Sustained SD + AV	3,134	2,720
		**Pre-emptive vaccination**	**Reactive vaccination**
**Scenario 4**: pandemic with virus which matches pre-emptive vaccine with efficacy of 75%	none	3,499	9*
	8 weeks SD + AV	3,507	1,219
	Sustained SD + AV	3,510	2,720

*indicates life years saved (LYS) value not statistically significantly different from zero due to stochastic simulation variation.

**Table 3:** Total cost of vaccination strategies for moderate pandemics (R = 1.9 and CFR = 0.25%) and severe pandemics (R = 2.7 and CFR = 1.5%)

	**Moderate pandemics (R = 1.9 and CFR = 0.25%)**		
**Scenarios**	**SD and AV intervention**	**Total cost ($) per 10,000 population**	
		**Pre-emptive vaccination**	**Reactive vaccination**
**Scenario 1**: no pandemic	none	$2,430,000	$0
		**Pre-emptive + Reactive vaccination**	**Reactive vaccination**
**Scenario 2**: pandemic with virus which does not match pre-emptive vaccine i.e. vaccine efficacy of 0%	none	$6,510,000	$4,080,000
	8 weeks SD + AV	$7,510,000	$5,080,000
	Sustained SD + AV	$10,460,000	$8,030,000
Unmitigated pandemic cost (no intervention)		$3,200,000	
		**Pre-emptive vaccination**	**Reactive vaccination**
**Scenario 3**: pandemic with virus which matches pre-emptive vaccine with efficacy of 30%	none	$4,250,000	$4,080,000
	8 weeks SD + AV	$5,020,000	$5,080,000
	Sustained SD + AV	$6,820,000	$8,030,000
Unmitigated pandemic cost (no intervention)		$3,200,000	
		**Pre-emptive Vaccination**	**Reactive vaccination**
**Scenario 4**: pandemic with virus which matches pre-emptive vaccine with efficacy of 75%	none	$3,630,000	$4,080,000
	8 weeks SD + AV	$4,800,000	$5,080,000
	Sustained SD + AV	$6,310,000	$8,030,000
Unmitigated pandemic cost (no intervention)		$3,200,000	
	**Extreme pandemics (R = 2.7 and CFR = 1.5%)**		
**Scenarios**	**SD and AV intervention**	**Total cost ($) per 10,000 population**	
		**Pre-emptive vaccination**	**Reactive vaccination**
**Scenario 1**: no pandemic	none	$2,430,000	$0
		**Pre-emptive + Reactive vaccination**	**Reactive vaccination**
**Scenario 2**: pandemic with virus which does not match pre-emptive vaccine i.e. vaccine efficacy of 0%	none	$17,480,000	$15,050,000
	8 weeks SD + AV	$15,000,000	$12,570,000
	Sustained SD + AV	$13,430,000	$11,000,000
Unmitigated pandemic cost (no intervention)		$14,180,000	
		**Pre-emptive vaccination**	**Reactive vaccination**
**Scenario 3**: pandemic with virus which matches pre-emptive vaccine with efficacy of 30%	none	$10,190,000	$15,050,000
	8 weeks SD + AV	$8,420,000	$12,570,000
	Sustained SD + AV	$8,810,000	$11,000,000
Unmitigated pandemic cost (no intervention)		$14,180,000	
		**Pre-emptive vaccination**	**Reactive vaccination**
**Scenario 4**: pandemic with virus which matches pre-emptive vaccine with efficacy of 75%	none	$3,760,000	$15,050,000
	8 weeks SD + AV	$5,100,000	$12,570,000
	Sustained SD + AV	$6,970,000	$11,000,000
Unmitigated pandemic cost (no intervention)		$14,180,000	

**Table 4:** Cost effectiveness (cost per life years saved) of vaccination strategies for moderate pandemics (R = 1.9 and CFR = 0.25%) and severe pandemics (R = 2.7 and CFR = 1.5%)

	**Moderate pandemics (R = 1.9 and CFR = 0.25%)**		
**Scenarios**	**SD and AV intervention**	**Cost ($) per LYS**	
		**Pre-emptive vaccination**	**Reactive vaccination**
**Scenario 1**: no pandemic occurs	none	-	-
		**Pre-emptive + Reactive vaccination**	**Reactive vaccination**
**Scenario 2**: pandemic with virus which does not match pre-emptive vaccine i.e. vaccine efficacy of 0%	none	$1,627,500	$1,020,000
	8 weeks SD + AV	$29,684	$20,079
	Sustained SD + AV	$24,846	$19,074
		**Pre-emptive vaccination**	**Reactive vaccination**
**Scenario 3**: pandemic with virus which matches pre-emptive vaccine with efficacy of 30%	none	$13,622	$1,020,000
	8 weeks SD + AV	$12,096	$20,079
	Sustained SD + AV	$15,897	$19,074
		**Pre-emptive** **vaccination**	**Reactive vaccination**
**Scenario 4**: pandemic with virus which matches pre-emptive vaccine with efficacy of 75%	none	$8,013	$1,020,000
	8 weeks SD + AV	$10,435	$20,079
	Sustained SD + AV	$13,658	$19,074
	**Extreme pandemics (R = 2.7 and CFR = 1.5%)**		
**Scenarios**	**SD and AV intervention**	**Cost ($) per LYS**	
		**Pre-emptive vaccination**	**Reactive vaccination**
**Scenario 1**: no pandemic occurs	none	-	-
		**Pre-emptive + Reactive vaccination**	**Reactive vaccination**
**Scenario 2**: pandemic with virus which does not match pre-emptive vaccine i.e. vaccine efficacy of 0%	none	$1,942,222	$1,672,222
	8 weeks SD + AV	$12,305	$10,312
	Sustained SD + AV	$4,938	$4,044
		**Pre-emptive vaccination**	**Reactive vaccination**
**Scenario 3**: pandemic with virus which matches pre-emptive vaccine with efficacy of 30%	none	$6,066	$1,672,222
	8 weeks SD + AV	$3,097	$10,312
	Sustained SD + AV	$2,811	$4,044
		**Pre-emptive vaccination**	**Reactive vaccination**
**Scenario 4**: pandemic with virus which matches pre-emptive vaccine with efficacy of 75%	one	$1,075	$1,672,222
	8 weeks SD + AV	$1,454	$10,312
	Sustained SD + AV	$1,986	$4,044

### Reactive vaccination

A comprehensive cost effectiveness analysis of reactive vaccination including 6 months of development delay has been conducted in [15]. Reactive vaccination alone is an ineffective strategy in saving lives and not cost effective (see Tables 2 and 4, reactive vaccination column and Figure 2), since vaccination occurs well after the pandemic peak and has essentially no effect. The addition of at least 8 weeks social distancing and antiviral interventions to the reactive vaccination greatly increases effectiveness and cost effectiveness (see Tables 2 and 4). Sustained SD plus AV further improve effectiveness and cost effectiveness for both moderate and severe pandemics (see Tables 2 and 4, reactive vaccination). For severe pandemics, the addition of sustained SD and AV to reactive vaccination improves the relative cost effectiveness by decreasing total pandemic costs and increasing the number of life year saved. For moderate pandemics, it is the most cost effective strategy but it also increases the total pandemic costs (see Tables 3 and 4, reactive vaccination).

### Scenario 1: no pandemic occurs

One of the main assumptions of this study is that a future pandemic will occur in a 30 year time frame. If no pandemic occurs, obviously there is no need for reactive vaccines. Therefore, reactive vaccination policy where no vaccine is actually developed costs nothing, while the pre-emptive vaccination policy that costs approximately $2.5 million per 10,000 population (see Table 3 – scenario 1). This scenario is has been included essentially to quantify the direct cost incurred by pre-emptive vaccination. Note that in this scenario cost effectiveness is undefined, as no lives are lost due to the pandemic or saved due to interventions.

### Scenario 2: pandemic virus strain does not match pre-pandemic vaccines i.e. vaccine efficacy of 0%

In this scenario, it was assumed that the ineffectiveness of the pre-pandemic vaccine would be quickly recognised, and a reactive vaccine would be developed and deployed to mitigate the effect of the pandemics. The effectiveness and cost effectiveness results, for both moderate and severe pandemics, are as for the reactive strategy, except that under the pre-emptive vaccination policy the additional cost of the pre-pandemic vaccine is paid, making the pre-emptive policy less cost effective (see Tables 2, 3 and 4 for scenario 2). For example, the relative cost effectiveness ratio of the pre-emptive policy is approximately $25,000/LYS whereas $19,000/LYS for the reactive policy, for moderate pandemics (see Table 4 for scenario 2). Although these results are unsurprising, quantification of the additional cost of having to develop and use both vaccines allows us to determine how the cost-effectiveness of a pre-emptive vaccination policy degrades with increasing probability of vaccine failure.

### Scenario 3: pre-pandemic vaccine matches pandemic virus with efficacy of 30%

#### Moderate pandemics

For moderate pandemics, the results suggested that pre-emptive vaccination strategies with or without social distancing and antivirals were more effective, less costly and more cost effective compared to their reactive vaccination counterparts (see Tables 2, 3, 4 and Figure 2 – centre panel, green-diamond and green-cross). This is because for moderate pandemics, the cohort of immune individuals created by vaccination reduces the effective reproduction of the pandemic and allows limited duration social distancing and antiviral measures to be highly effective.

The most cost effective strategy was pre-emptive vaccination coupled with 8 weeks SD plus AV. This resulted the cost effectiveness ratio of $12,000 per LYS while saving approximately 415 life years per 10,000 population, whereas the reactive counterpart resulted $20,000 per LYS while saving approximately 253 life years per 10,000 population (see Table 4 for moderate pandemics). The most effective strategy was the pre-emptive vaccination combined with *sustained* SD plus AV, which saved approximately 430 life years per 10,000 population. This was the most costly pre-emptive strategy however, costing approximately $6.8 million per 10,000 population, because its sustained social distancing interventions incurred additional costs (see Figure 3 – centre panel, moderate pandemics) while saving relatively few additional lives.

### Severe pandemics

For severe pandemics, the results indicated that the most effective and cost effective strategies included pre-emptive vaccination combined with sustained SD plus AV. This strategy is shown in Tables 2, 4 and Figure 2 – centre panel (labelled as P + SSD red-diamond for severe pandemics).

This strategy was highly cost effective due to the fact that with a high CFR, prevention of infection translated into prevented hospitalisation, ICU usage and death. Thus, all interventions reduced both life years lost and costs, rendering tem highly cost effective. This can be seen in Figure 3 – scenario 3 for severe pandemics, which presents the break-down of cost components for each intervention strategy and scenario. Due to this combined effect, this strategy could save approximately 3100 life years per 10,000 population, which was the highest number of life years saved, and cost approximately $2,800 per LYS, which was the lowest cost effectiveness ratio among the modelled interventions (see Tables 2 and 4).

The next most cost effective strategy was that which combined pre-emptive vaccination with 8 weeks SD plus AV (see Table 4 and Figure 2 centre panel – P + LSD red diamond). When compared to the reactive counterpart, the combined pre-emptive vaccination with 8 weeks SD plus AV is approximately 55% more effective and approximately 30% less costly (see Tables 2 and 3 for scenario 3). This is explained by the fact that in the case of the reactive vaccination policy the pandemic resurges once social distancing interventions are relaxed, whereas the usage of pre-pandemic vaccines creates a significant proportion of immune individuals which prevents the pandemic resurgence, resulting in a significant reduction in mortality and total cost.

### Scenario 4: pre-pandemic vaccine matches pandemic virus with efficacy of 75%

#### ***Moderate pandemics***

Under this scenario, the pre-emptive vaccination policy was highly effective and cost effective compared to the reactive vaccination policy. The most cost effective strategy was purely pre-emptive vaccination, resulting a cost per life year saved ratio of $8000. The addition of social distancing and antivirals incurred total costs resulting in $10,400/LYS for 8 weeks social distancing plus antivirals and $13,700/LYS for the sustained social distancing plus antivirals respectively. The additional social distancing and antiviral costs of these combined strategies incurs productivity losses due to social distancing interventions, without preventing many further symptomatic infections or deaths. This is apparent in Figure 3 – right panel, which shows the cost breakdown of different cost components.

For severe pandemics, the results for scenario 4 follow the same pattern as for moderate pandemics, but are more pronounced, since the highly effective intervention saves both lives and cost.

### Sensitivity analysis

Sensitivity analyses were conducted for key model parameters to determine how alternative assumptions about pre-emptive vaccination affected the relative cost effectiveness pre-emptive and reactive vaccination strategies.

We specifically examined pre-emptive vaccination coverage, the time between pre-pandemic vaccine renewals, the expected time between pandemics and the chance of pre-pandemic vaccine mismatch. Significant findings are summarised below. We further performed sensitivity analyses for lower/higher weekly wages found in EU [61] and including/excluding productivity losses due to illness. These sensitivity analyses are presented in Additional File 1. Further sensitivity analysis of key parameters related to reactive vaccination can be found in details in the supplementary information of [15].

### Vaccination coverage

**Figure 4:**
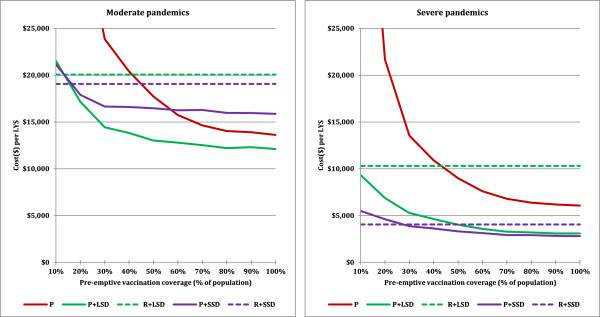
**Impact of vaccination coverage on the cost effectiveness.** Interventions strategies are labelled as Figure 2.

We assume that for a moderate or severe pandemic, having a CFR of 0.25% or greater, 100% or near 100% coverage is plausible for a reactive vaccination strategy. For pre-emptive vaccination, where there is no deadly pandemic underway to focus the attention of the public, it may not be possible to achieve a high level of vaccination coverage. We therefore extended our cost effectiveness analysis to vary pre-emptive vaccination coverage from 10% to 100%, for all scenarios and interventions considered in this study (see Figure 4).For moderate pandemics, pre-emptive vaccination with 20% coverage coupled with 8 weeks social distancing and antiviral interventions resulted in better cost effectiveness than the most cost effective reactive vaccination strategy; achieving 50% pre-emptive vaccination coverage was sufficient for near optimal (within 10%) cost effectiveness (see Figure 4 – left panel).For severe pandemics, pre-emptive vaccination with at least 30% coverage together with sustained social distancing and antivirals resulted in better cost effectiveness than the most cost effective reactive vaccination strategy, and 60% pre-emptive vaccination coverage was near optimal cost effectiveness (see Figure 4 – right panel).

### Vaccine renewal frequency

**Figure 5:**
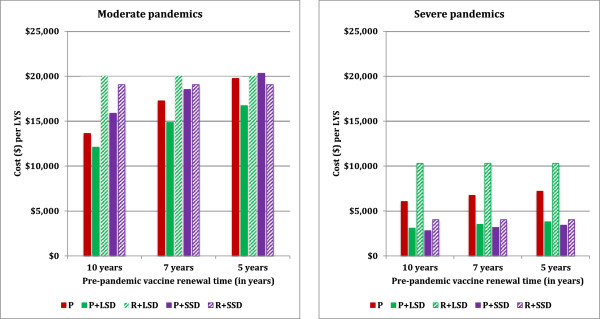
**Impact of vaccine renewal frequency on the cost effectiveness.** Interventions strategies are labelled as Figure 2.

Depending on the rate of emergence of new potential pandemic virus strains and the degree of cross-strain reactivity that can be achieved with a pre-emptive vaccine, vaccines may have to be renewed more frequently than the 10 year baseline value assumed in the main results. We alternatively assumed that the pre-pandemic vaccine would be renewed every 7 or 5 years, incurring new vaccine development and administration costs. The analyses suggested that more frequent renewal makes pre-emptive vaccination less cost effective; however for both moderate and severe pandemics the most cost effective strategy is still pre-emptive vaccination coupled social distancing, even if vaccine renewal time is reduced to 5 years (see Figure 5).

### Pandemic frequency

**Figure 6:**
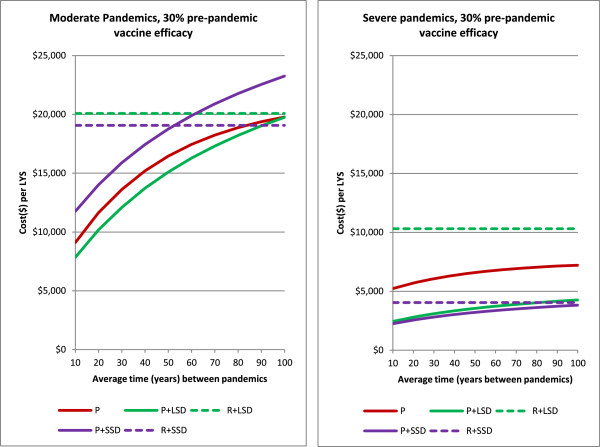
**Impact of pandemic frequency on the cost effectiveness.** Interventions strategies are labelled as Figure 2.

If pandemics occurred at a lower rate than the average of one every 30 years assumed in the main results, pre-emptive vaccination would become less cost effective, since the pre-emptive vaccine renewal program would result in more vaccines being developed per pandemic. We examined the sensitivity of the results to pandemic frequency by calculating costs and cost effectiveness assuming average time between pandemics ranging from 10 to 100 years (see Figure 6). It was found that pre-emptive vaccination (coupled with social distancing) remained the most cost-effective strategy for pandemic return times up to 80 years.

### Pre-pandemic vaccine mismatch

**Figure 7:**
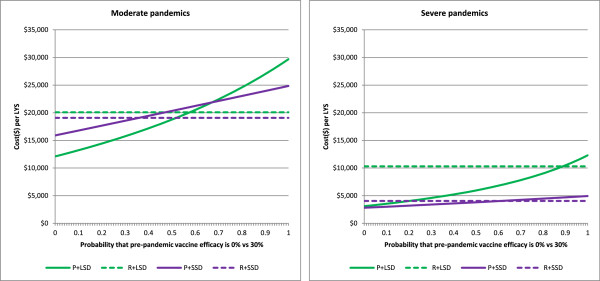
**Impact of pre-pandemic vaccine mismatch on the cost effectiveness.** Interventions strategies are labelled as Figure 2.

The vaccine effectiveness of a pre-pandemic vaccine is a major unknown: we have examined three scenarios of 0%, 30% and 75% effectiveness. However without probabilities attached to these scenarios, a quantitative assessment of cost-effectiveness in the face of uncertainty cannot be made. We conducted a simple analysis by assuming that VE would be 0% effective with some probability p, and 30% effective with probability (1-p). In other words, there is a probability of p of complete vaccine failure. Figure 7 shows the expected (in the technical sense) cost effectiveness as a function of p. This shows that the most cost effective pre-emptive vaccination strategy (including social distancing and antivirals) remains more cost effective than reactive strategies with a vaccine failure probability of 40% or lower.

## Discussion

The possible emergence of a highly pathogenic influenza pandemic presents a challenge to public health planning. Vaccination with an effective vaccine is an ideal solution; however such a vaccine would take at least 6 months to develop, test, produce and distribute with current proven techniques, by which time the pandemic is likely to have reached most parts of the world. Antiviral neuraminidase drugs and social distancing measures of sufficient rigor would be capable of supressing transmission, however these could be extremely expensive and possibly unsustainable [15,29,40,62].

A pre-pandemic vaccine, developed to provide cross-clade protection and administered prior to the pandemic, has the potential to address this problem. However this strategy comes with its own challenges: it is not certain what degree of vaccine efficacy that can be achieved against an unknown future influenza strain, it may be difficult to achieve high levels of vaccination coverage, the pre-pandemic would need to be periodically renewed and re-administered.

This modelling study has compared the relative effectiveness and cost effectiveness of reactive and pre-emptive vaccination strategies. In order to make a meaningful comparison, both strategies have been made as plausible as possible: the reactive strategy assumes a six month delay to vaccine availability; and the pre-emptive strategy assumes a lower vaccine efficacy and ongoing vaccine renewal costs. All other assumptions that are not related to vaccination have been kept the same for both vaccination policies, including the inclusion of various combinations social distancing and antiviral measures.

For moderate and severe influenza pandemics a policy of pre-pandemic vaccination combined with social distancing and antiviral interventions was found to be more effective and cost effective than a policy of reactive vaccination, provided that at least 30% of the population can be pre-vaccinated. While a pre-pandemic vaccine is presumed to have a lower efficacy than a matched reactive vaccine, pre-vaccination creates a cohort of individuals who are immune at the start of the pandemics. For moderate pandemics this reduces the effective reproduction of the pandemic and allows limited duration social distancing and antiviral measures to be highly effective. For severe pandemics, the infection avoided by pre-vaccination reduces both the number of lives lost and, due to avoided medical costs, the total cost of the pandemic.

The most effective and cost effective strategies for both reactive and pre-emptive vaccination involved the combination of vaccination with social distancing and antiviral measures. For reactive vaccination, sustained social distancing and antiviral measures (lasting for 6 months) was always most cost effective, since without some early intervention the pandemic peaked and subsided prior to the vaccination programme.

For pre-emptive vaccination the optimally cost-effective duration of social distancing depended on the pandemic severity: for severe pandemics sustained social distancing was the most cost effective, while for moderate pandemics 8 weeks of social distancing was more cost effective than sustained social distancing. In this case the presence of immune individuals, and the use of optimally timed [38] social distancing for 2 months was highly effective at reducing attack rate and mortality, to such a degree that additional months of social distancing did not significantly further reduce the attack rate.

If it transpired that the pre-pandemic vaccine was highly effective (VE 70%), the cohort of immune individuals was sufficient to prevent the pandemic, and pre-vaccination alone was then highly cost effective.

### Sensitivity analysis

Pre-emptive vaccination is an untried strategy, and while we have chosen plausible parameter values to represent this strategy in our model, it is possible that there are other plausible assumptions that might lead to a different assessment of cost effectiveness. In fact, our sensitivity analysis indicates that the results are quite robust to variation in the most important of these assumptions.

Unlike a reactive vaccination program, which we assume will be able to achieve high vaccination coverage during a highly pathogenic pandemic, it is possible that vaccination coverage of a pre-pandemic vaccination programme will not be able to achieve high levels of coverage. We found that pre-emptive strategies remained more cost effective compared to corresponding reactive strategies for pre-emptive vaccination coverage levels down to 30% (while reactive strategies remain at 100% coverage). Pre-vaccination also remained more cost-effective than reactive vaccination if pandemics were assumed to occur on average every 80 years, rather than the baseline assumption of 30 years; or if pandemic vaccines needed to be renewed every 5 years rather than the baseline assumption of every decade. Our sensitivity analysis also showed that if a policy of pre-emptive vaccination is backed up by reactive vaccination in the case of pre-pandemic vaccine failure (i.e. our scenario 2 results), the pre-emptive policy can tolerate a vaccine failure rate of 30% and remain competitive with a reactive-only policy.

Our economic analysis was conducted from a societal perspective, and includes costs due to productivity losses stemming from illness and social distancing interventions. Analysing the results from a health-care provider perspective and omitting these costs, we find that the most cost effective strategy as judged by the lowest cost per LYS ratio remains the same (results shown in Additional File 1).

As with all modelling studies, the numerical results presented in this study depend on assumptions and model parameters which are not known exactly. However, the results and trends discussed highlight the relative cost effectiveness of alternative intervention strategies – in this case, pre-emptive versus reactive vaccination strategies – that differ only on intervention parameters, and for which all the other model parameters are the same. These results should be robust to plausible variations in other model parameter values, as such variations in model parameter values will influence both simulation outcomes same way.

### Related research

Reactive vaccination strategies including vaccine development and deployment delay has been well studied in [15,16,32,35]. However, while no studies to our knowledge have explicitly compared the cost effectiveness of pre-emptive and delayed reactive strategies, several studies have considered strategies involving vaccination prior to or at the beginning of a pandemic with a vaccine of limited efficacy.

The study of Newall *et al*. [31] explicitly modelled pharmaceutical interventions for a severe pandemic, explicitly taking into account a 6-month delay before the use of a matched vaccine. This included scenarios where a lower efficacy stockpiled pre-pandemic vaccine was used at the beginning of the pandemic in addition to a matched vaccine after the delay. Although differences in assumptions mean that the study of Newall *et al*. is not directly comparable to the current study, it did show that sufficient coverage with a low-efficacy pre-pandemic vaccine could effectively mitigate the impact of a severe pandemic, and like the current study it concluded that cost effectiveness depended upon pandemic severity, pandemic frequency, vaccination coverage, and probability of pre-pandemic vaccine strain mismatch.

Several studies have included pre-vaccination in a cost-effectiveness analysis of pandemic influenza interventions [16,29,30,34]. While these studies did not compare pre-vaccination to vaccination strategies with a high-efficacy vaccine and a long delay, they did consider the use of limited efficacy vaccines at the beginning of a pandemic. Each of these studies found that with sufficient coverage, which depended upon vaccine efficacy and the use complementary social distancing and antiviral measures, limited efficacy vaccines could cost-effectively mitigate a pandemic.

### Limitations and further research

We recognise a number of limitations of the current study that suggest avenues for further research. While our univariate sensitivity analyses examined the effect of key unknowns, a probabilistic analysis could estimate cost-effectiveness while taking into account all plausible combination of unknowns. Such a multivariate analysis should define a (joint) probability distribution over pandemic transmissibility and severity, pandemic frequency, pre-pandemic vaccine efficacy, and actual vaccination coverage achieved; the expected effectiveness and cost effectiveness of intervention strategies could then be estimated by sampling from this distribution.

We have assumed that pre-pandemic vaccination would occur prior to a pandemic, perhaps by the inclusion of a pre-pandemic vaccine in a seasonal influenza vaccine. As noted previously, it may be difficult to achieve high levels of coverage in this way, given the relatively low uptake of influenza vaccines [63]. An alternative strategy might be to manufacture and keep a large stockpile of pre-pandemic vaccine, and to vaccinate at the appearance of a highly pathogenic pandemic. This may enable higher vaccination coverage, but would incur additional costs as vaccines would have to be stored and continually renewed due to limited shelf life. This strategy has been assessed in [30,34,64], but performing this analysis in the framework of the current study would allow a pre-pandemic stockpiled strategy it to be compared to the pre-emptive and reactive strategies.

The current study focuses on using mass vaccination to achieve a population-level reduction in transmission, with consequent reduction in attack rates, serious illness and death. As such we have assumed that when less than 100% vaccine coverage occurs, a *transmitters-first* strategy is adopted were vaccine is targeted towards those age groups most responsible for transmission (children and young adults). Previous modelling studies have indicated that an alternative *vulnerable first* strategy targeting groups at risk of serious illness and death due to influenza can be more cost-effective than the transmitters first strategy if vaccination is delayed relative to the start of the pandemic [28,35,56]. Since our reactive strategy is delayed, this might seem to indicate that we should simulate vulnerable-first. However our reactive strategy incorporates additional non-vaccination interventions during the delay period which slow or arrest pandemic spread, which is not the case in the other studies examining delayed vaccination. Since our previous study addressing the combination of delayed vaccination and additional interim measures showed that transmitters first vaccination was effective, we have retained this assumption in the current study [15]. We also note that the optimal choice between a *transmitters-first* and *vulnerable-first* can also depend upon population demographics and the presence of pre-existing immunity [35].

We have considered influenza pandemics of severity category 2 and up on the CDC scale (i.e. with CFR > =0.25%). This corresponds to the 1918, 1956, and 1968 pandemics, but not the 2009 pandemic. This focus was motivated by previous studies that indicated that reactive vaccination was unlikely to be cost effective [15,31].

Finally, the demographic, economic and health care assumptions of our simulation model are based on Australian population characteristics. However, the results of the studies that use our simulation model [36] are consistent with a variety of other individual-based simulation models at a variety of scales (e.g. small community [65-67], country [68,69]), in as far as comparable pandemics and intervention strategies are being evaluated. We thus believe that the model is broadly representative of developed world cities: sensitivity analyses included in this study showed that the total cost of a pandemic varies directly with income levels, since illness-related productivity losses make up a substantial part of the pandemic cost, but that the pattern of relative cost/LYS ratio between strategies is insensitive to a wide range of income levels.

## Conclusion

This study has quantified the effectiveness and cost effectiveness of plausible and comparable pre-emptive and reactive vaccination strategies, with and without accompanying social distancing and antiviral interventions. We found that provided a sufficient level of vaccination coverage can be achieved (approximately 30% in the case of a severe pandemic), pre-pandemic vaccination strategies have the potential to be more effective and cost-effective and reactive strategies where a vaccine is developed and deployed after the start of a pandemic. One clear risk of a pre-pandemic strategy is that if a future pandemic were to be of a subtype completely mismatched with respect to the pre-pandemic vaccine, the upfront cost of development and production of the pre-pandemic vaccine would be wasted. However, if the probability of vaccine failure can be kept sufficiently low, a policy of pre-vaccination with back-up reactive vaccination in case of vaccine failure can still be more cost-effective than a policy of reactive vaccination alone. Our analysis includes the assumption that pre-pandemic vaccines would be continually renewed in order to track the strains most likely to present a pandemic influenza threat.

Another key finding is that non-vaccination interventions, including antiviral treatment and prophylaxis, and social distancing measures such as school closure, are complementary to vaccination, improving the effectiveness and cost effectiveness of both pre-emptive and reactive vaccination.

Reactive vaccination strategies exist which are as effective at mortality reduction as pre-emptive strategies, though less cost effective. However these strategies require vaccination to be preceded by the use of rigorous social distancing interventions, including strict school closure and community contact reduction, and this would need to be sustained for many months, resulting in a high economic cost and great social disruption.

## Competing interests

The authors declare that they have no competing interests.

## Authors’ contributions

GM, JK and NH were responsible for the conception and design of the simulation experiments. NH and JK were responsible for simulation and economic model implementation. NH conducted simulation experiments. All authors were involved in the analysis of simulation results and writing the manuscripts. All authors read and approved the final manuscript.

## Pre-publication history

The pre-publication history for this paper can be accessed here:

http://www.biomedcentral.com/1471-2334/14/266/prepub

## Supplementary Material

Additional file 1 A model-based economic analysis of pre-pandemic influenza vaccination cost effectiveness. Click here for file
