# Supplementary material for: A model-based economic analysis of pre-pandemic influenza vaccination cost-effectiveness
Source: BMC Infect Dis. 2014 May 16;14:266. doi: 10.1186/1471-2334-14-266 (PMC4045999; doi:10.1186/1471-2334-14-266)
Supplement: Additional file 1 — A model-based economic analysis of pre-pandemic influenza vaccination cost effectiveness. [file 1471-2334-14-266-S1.docx]

**A model-based economic analysis of pre-pandemic influenza vaccination cost effectiveness – Additional File 1**

Nilimesh Halder^1^, Joel K Kelso^1*^, and George J Milne^1^

^1^School of Computer Science and Software Engineering, University of Western Australia, Stirling Highway, Crawley, Western Australia 6009, Australia

Emails: [nilimesh.halder@uwa.edu.au](mailto:nilimesh.halder@uwa.edu.au), [joel.kelso@uwa.edu.au](mailto:joel.kelso@uwa.edu.au), [george.milne@uwa.edu.au](mailto:george.milne@uwa.edu.au)

*corresponding author

**Simulation Model Description**

**Population contact network**

The simulation model captures the contact dynamics of the population of Albany, Western Australia using census and state and local government data [1], allowing us to replicate the individual age and household structure of all households in this town of approximately 30,000 individuals. Human contact networks were modelled as a network of connected households and contact hubs such as schools, childcare centres, workplaces and a regional hospital. A conceptual framework of human contact networks was presented in Figure A1.1. Individuals in each household and hub made contacts within a close contact mixing group, taken to be the entire household or a subset of larger hubs, and also made additional non-hub based random contacts in the wider community. Using this community-based population model, we conducted stochastic, individual-based spatial simulations of an influenza epidemic. We assumed that an average of one new infection per day was stochastically introduced into the population during the whole period of the simulations. The simulation period was divided into 12 hour day/night cycles and during each simulation cycle a nominal location of each individual was determined; taking into consideration the cycle type (day/night, weekday/weekend), infection state of each individual and whether child supervision was needed to look after a child at home. Individuals occupying the same location during the same time period (cycle) were assumed to come into potential infective contact.

**Influenza transmission model**

In the simulation model we assumed that infectious transmission could occur when an infectious and susceptible individual came into contact during a simulation cycle. The number of contacts made by each individual each day in school, work and community settings were adjusted to reproduce the proportion of cases occurring in different settings as reported by empirical studies, specifically 40% of infections occurred in households, 30% in schools and workplaces, and 30% in the wider community [[8,](#_ENREF_2)57,58]. Contacts within schools and workplaces occurred in fixed-size mixing groups of maximum size 10; within mixing groups contact was assumed to be homogeneous. Community contacts occurred between randomly selected individuals, weighted toward pairs of individuals with nearby households. The mixing group sizes, and location-specific distribution of where infection occurs, are given in in Tables A1.1 and A1.2 respectively. Age specific infection rates were also shown in Figure A1.2.

Following each contact a new infection state for the susceptible individual (either to remain susceptible or to become infected) was randomly chosen via a Bernoulli trail [[2](#_ENREF_5)]. Once infected an individual progressed through a series of infection states according to a fixed timeline.

The probability that a susceptible individual would be infected by an infectious individual was calculated according to the following transmission function, which takes into account the disease infectivity of the infectious individual *I_i_* and the susceptibility of susceptible individual *I_s_* at the time of contact.

*P_trans_*(*I_i_*,*I_s_*) = β × *Inf*(*I_i_*) × *Susc*(*I_s_*) × *AVF*(*I_i_*,*I_s_*) × *Vaccine*(*I_s_*)

The baseline transmission coefficient β was initially chosen to give an epidemic with a final attack rate of 17.4% which is consistent with seasonal influenza as estimated in Table 3 of [3]. To achieve simulations under a range of reproductive numbers, β was increased from this baseline value to achieve epidemics of various R_0_ magnitudes; details of the procedure for estimating β and R_0_ are given in [4].

The disease infectivity parameter *Inf*(*I_i_*) was set to 1 for symptomatic individuals at the peak period of infection and then to 0.5 for the rest of the infectivity period The infectiousness of asymptomatic individuals is also assumed to be 0.5 and this applies to all infected individuals after the latent period but before onset of symptoms. The infection profile of a *symptomatic* individual was assumed to last for 6 days as follows: a 0.5 day latent period (with *Inf*(*I_i_*) set to 0) is followed by 1 day asymptomatic and infectious, where *Inf*(*I_i_*) is set to 0.5; then 2 days at peak infectiousness (with *Inf*(*I_i_*) set to 1.0); followed by 2.5 days reduced infectiousness (with *Inf*(*I_i_*) set to 0.5). For an infected but *asymptomatic* individual the whole infectious period (of 5.5 days) is at the reduced level of infectiousness with *Inf*(*I_i_*) set to 0.5. This infectivity profile is a simplification of the infectivity distribution found in a study of viral shedding [5]. As reported below in the results section for the unmitigated no intervention scenario, these assumptions regarding the duration of latent and infectious periods lead to a mean generation time (serial interval) of 2.47 days which is consistent with that estimated for A/H1N1 2009 influenza [6].

Following infection an individual is assumed to be immune to re-infection for the duration of the simulation. We further assume that influenza symptoms develop one day into the infectious period [5], with 20% of infections being asymptomatic among children and 32% being asymptomatic among adults. These percentages were derived by summing the age-specific antibody titres determined in [7]. Symptomatic individuals will withdraw into the home with the following probabilities; adults 50% and children 90%, which is in keeping with the work of [8, 9].

The susceptibility parameter *Susc*(*I_s_*) is a function directly dependent on the age of the susceptible individual. It captures age-varying susceptibility to transmission due to either partial prior immunity or age-related differences in contact behaviour. To achieve a realistic age specific infection rate, the age-specific susceptibility parameters were calibrated against the serologic infection rates for seasonal H3N2 in 1977-1978 in Tecumseh, Michigan [3] . Details of this calibration process may be found in [4].

The antiviral efficacy factor *AVF*(*I_i_*,*I_s_*) **=** (1 - *AVE_i_*)*(1 - *AVE_s_*) represents the potential reduction in infectiousness of an infected individual (denoted by *AVE_i_*) induced by antiviral treatment, and the reduction in susceptibility of a susceptible individual (denoted by *AVE_s_*) induced by antiviral prophylaxis. When no antiviral intervention was administrated the values of both *AVE_i_* and *AVE_s_* were assumed to be 0, indicating no reduction in infectiousness or susceptibility. However, when antiviral treatment was being applied to the infectious individual the value of *AVE_i_* was set at 0.66, capturing a reduction in infectiousness by factor of 66% [10]. Similarly, when the susceptible individual was undergoing antiviral prophylaxis the value of *AVE_s_* was set to 0.85 indicating a reduction in susceptibility by a factor of 85% [10]. This estimate is higher than most previous modelling studies, which assume an *AVE_s_* of 30% (e.g. [8, 11, 12]). This common assumption appears to stem from an estimate made in [13] based on 1998-1999 trial data. Our higher value is based on a more comprehensive estimation process reported in [10], which also incorporated an data from an additional study performed in 2000-2001 [14]. It is also in line with estimates of 64%-89% reported in [15].

**Vaccination model**

The vaccination parameter *Vaccine*(*I_s_*) represents the potential reduction in susceptibility owing to vaccination, representing some level of immunity. For unvaccinated individuals, or for individuals for whom the vaccine is ineffective, this parameter is 1.0, indicating no reduction in susceptibility. For effectively vaccinated individuals, the parameter value changed from 1.0 to 0.0 according to a schedule described below.

A two-dose pre-emptive vaccination strategy is considered, as trials of candidate pre-pandemic H5N1 vaccines indicate the need for a two-dose regime to induce immunity [16-22]. It is assumed that pre-pandemic vaccination would be an ongoing process, possibly as a component vaccine of a multivalent seasonal influenza vaccine. It is also assumed that the time between pandemics is 30 years and that pre-pandemic vaccines were updated every ten years to reflect which influenza strains circulation in wildfowl and poultry populations give most concern from a zoonotic perspective, such as H5N1 and H7N9, for example. Trials of candidate pre-pandemic vaccines for the H5N1 influenza virus have shown seroconversion rates (defined as having a fourfold neutralizing seroconversion rate) between 60 and 90 per cent [17,19-21]. However, these pre-pandemic vaccines may not be closely matched to an emergent influenza strain or may offer only limited cross-strain protection within the virus subtype, thus an efficacy of 30% is assumed. For completeness, a high efficacy pre-pandemic vaccine which closely matches the virus subtype with 75% efficacy was also considered, and an assumption of full vaccination coverage is also made. We further assumed that reduced vaccine efficacy in elderly people as mentioned in [23][60].

A two-dose reactive vaccination strategy is considered for moderate and severe pandemics, assuming that individuals are naïve to a future influenza strain and that a two-dose vaccine is essential to achieve immunity [21,22]. A single dose vaccine was reactively used during the H1N1 2009 pandemic [59]. Therefore, we modelled a single dose reactive vaccination strategy in this study for a mild pandemic with transmissibility and pathogenicity similar to the H1N1 2009 pandemic [6,42,59]. During the H1N1 2009 pandemic the first supplies of a suitable vaccine became available after 5-6 months following the appearance of the new strain of H1N1 influenza. In this study, a 6 months delay from the onset of the pandemic to the initiation of a vaccine campaign is assumed, as is a vaccination rate of 1% of the population per day and availability of a highly effective vaccine is assumed. Trials of candidate vaccines for the H1N1 2009 pandemic influenza showed seroconversion rates of vaccines between 82 and 92 per cent [25]; vaccines with an efficacy of 75% are therefore assumed with a lower efficacy in elderly population [23][60]. It is also assumed that the vaccination campaign will continue until the local epidemic effectively ceases, by creating a cohort of vaccine immune individuals. An assumption of full vaccination coverage is further made. It was assumed that vaccination was prioritised so that age groups known to have higher transmission rates would be vaccinated first. Previous modelling results have indicated that a transmitters-first vaccination strategy is more effective in reducing both attack and mortality rates than a vulnerable-first approach when wide vaccination coverage is possible [24, 26].For those where vaccination failed the vaccine has no effect. It was assumed that vaccinated individuals who failed to develop immunity were no less infectious than unvaccinated individuals. It may be the case that vaccinated individuals who subsequently contract influenza experience a less severe infection, which may reduce morbidity and infectiousness. This assumption was not modelled, hence the results are if anything somewhat conservative.

Moreover, it was assumed complete immunity would only be achieved (for those whom vaccination was successful) after 2 doses of a vaccine. In the absence of definitive data, the conservative assumption was made that an individual would not develop any humoral immunity in the week immediately following the first vaccine dose. It was further assumed that, in the proportion of the population destined to achieve full immunity, protection from infection would rise in a linear fashion from zero at 1 week to 30% at 3 weeks, after the first vaccine dose. Further details and rationale for this immunity model are given in [24], along with sensitivity analyses on key assumptions.

We assumed that full immunity developed 1 week after the second vaccine dose and modelled this immunity rising in a linear fashion from week 3 to week 4. This one week time scale is based on of rapid immune response (seroconversion within 7 days) after doses of booster vaccines [27].

**Social Distancing and Antiviral Strategies**

We examined a range of social distancing and antiviral intervention strategies including school closure, antiviral drugs for treatment and prophylaxis, and community contact reduction. These interventions were considered in combination with vaccination, and social distancing interventions were considered for either sustained periods or periods of 8 weeks for moderate and severe pandemics. Sustained periods of social distancing may not be plausible for a mild pandemic since such rigorous interventions were not considered during the H1N1 2009 pandemic [59]. Therefore, 2 weeks and 8 weeks of social distancing were considered for the mild pandemic scenario.

Antiviral drug interventions and social distancing interventions were initiated when specific threshold numbers of symptomatic individuals were diagnosed in the community, and this triggered health authorities to mandate the intervention response. This threshold was taken to be 0.1% of the population. It was assumed that 50% of all symptomatic individuals were diagnosed, and that this diagnosis occurred at the time symptoms appeared.

For sustained school closure, all schools were closed simultaneously once the intervention trigger threshold was reached. For fixed duration school closure, schools were closed individually as follows: for a primary school the whole school was closed if 1 or more cases were detected in the school; in a high school only the class members of the affected class were isolated (sent home and isolated at home) if no more than 2 cases were diagnosed in a single class; however if there were more than 2 cases diagnosed in the entire high school the school was closed. Note that these school closure policies were only activated after the community-wide diagnosed case threshold was reached; cases occurring in schools before this time did not result in school closure. This policy of triggering school closure based on epidemic progression avoids premature school closure which can reduce the effectiveness of limited duration school closure [28]. Community contact reduction (CCR) was modelled by assuming that on days when the intervention was in effect all individuals made 50% fewer random community contacts.

Antiviral drugs used for treatment of symptomatic cases, plus prophylaxis of all household members of a symptomatic case were modelled. It was assumed that 50% of symptomatic individuals would be identified for antiviral treatment and/or prophylaxis, and that treatment and prophylaxis would occur 24 hours after the appearance of symptoms. It was assumed that an individual would receive at most one prophylactic course of antiviral drugs. Further details of antiviral interventions along with sensitivity analyses to key assumptions are given in [29, 30].

**Costs and Economic Analysis**

The economic model translates the age-specific infection profile of each individual in the modelled population, as derived by the Albany simulation model, into the overall pandemic cost burden. This overall cost comprises the following components: costs arising directly from interventions including productivity losses due to social distancing, antiviral costs and vaccination costs (this vaccination cost includes the cost of a vaccine itself, delivery cost of vaccines, and time and travel cost required to obtain vaccines); loss of productivity in the workplace arising from illness; medical costs associated with hospitalisation and GP visits of ill individuals; and productivity losses due to death. In main results in the paper, the overall total costs without productivity losses due to death were presented. A full set of additional results that includes the productivity losses due to death was given in this additional file for the completeness.

Age-specific hospitalisation costs are achieved by multiplying the average cost per day by average length of stay for each age group [31, 32]. Hospitalisation costs, including ICU costs, those involving medical practitioner visits, and antiviral drug (and their administration) costs and vaccination costs are taken from the literature [33-35]. Treatment costs, lengths of stay in hospital (both ICU and non-ICU), and other cost data used in establishing the overall cost of mitigated and unmitigated epidemics in the modelled community.

Productivity losses due to illness and interventions (e.g. necessary child-care due to school closure and workforce reduction) were obtained using average wages and average work-days lost; the latter being determined from day-to-day outbreak data generated by the simulation model. Assumed average wages are taken from the literature [36, 37].

Indirect production losses due to death were derived using a human capital method, based on the net present value of future earnings for an average age person in each age group. This was calculated by multiplying the age-specific number of deaths due to illness by the average expectancy in years of future earnings of an individual by an average annual income [37]. We assumed a maximum earning period up to age 65. Productivity losses due to death were discounted at 3% annually, which is a standard discount used to express all future income in present values [38].

All costs are reported in 2012 US dollars using consumer price index adjustments [39]. 2012 US dollar values are used to make the results readily convertible to a wide range of developed countries.

**Table A1.1: Model parameters values and cost data**

| **Parameters** | **Values (sensitivity analysis values)** | **Source** |
| --- | --- | --- |
| **Pandemic Scenarios** |  |  |
| Mild pandemic | R = 1.5, Attack Rate = 14%; CFR = 0.03% | [[6](#_ENREF_15), [40-42](#_ENREF_36)] |
| Moderate pandemic | R = 1·9, Attack Rate = 33%; CFR = 0·25% | [[13](#_ENREF_15), [43-46](#_ENREF_36)] |
| Severe pandemic | R = 2.7, Attack Rate = 44%; CFR = 1.5% | [[44-50](#_ENREF_37)] |
| Average hospital stay (days) | 4 | [[51](#_ENREF_44), [52](#_ENREF_45)] |
| Average ICU stay (days) | 7 | [[51](#_ENREF_44), [52](#_ENREF_45)] |
| **Interpersonal contact parameters** |  |  |
| School class mixing group size | 10 | [[8](#_ENREF_3), [58](#_ENREF_4)]* |
| Maximum workplace mixing group size | 10 | [[8](#_ENREF_3), [58](#_ENREF_4)]* |
| Number of community contacts per person per day | 4 | [[8](#_ENREF_3), [58](#_ENREF_4)]* |
| **Vaccination parameters** |  |  |
| Pre-emptive vaccine efficacy | 30% (10%, 20%) | [[53-55](#_ENREF_46)] |
| Reactive vaccine efficacy | 75% (65%, 85%) | [[53-55](#_ENREF_46)] |
| Vaccination coverage | 100% (10% - 100%) | - |
| **Antiviral Efficacy** |  |  |
| Infectiousness reduction | 66% | [[10](#_ENREF_12), [15](#_ENREF_17)] |
| Susceptibility reduction | 85% | [[10](#_ENREF_12), [15](#_ENREF_17)] |
| prophylaxis symptom reduction probability | 50% | [[10](#_ENREF_12)] |
| **Logistic parameters related to AVs** |  |  |
| Diagnosis delay | 24 h | - |
| Diagnosis ratio | 50% | - |
| Maximum antiviral courses given for treatment | 1 course per person for 5 days | - |
| Maximum antiviral courses given for prophylaxis | 1 course per person for 10 days | - |
| **Social Distancing Intervention Related Parameters** |  |  |
| School Closure Duration | 8 weeks and sustained | - |
| School Closure Trigger | 20-40 community cases | [[28](#_ENREF_21)] |
| Probability of a child withdrawal from school | 1·0 | - |
| Community Contact Reduction (CCR) Duration | 8 weeks and sustained | - |
| CCR trigger | 2 weeks after first case | - |
| CCR withdrawal probability | 0.5 | - |
| **Cost analysis assumptions** |  |  |
| Average wages (per week) | $882 | [[37](#_ENREF_30)] |
| Average school closure cost (per student per day) | $20.3 | [[36](#_ENREF_29)] |
| Average GP visit cost | $112.94 | [[35](#_ENREF_28)] |
| Average hospitalization cost (per day) | $1100 | [[35](#_ENREF_28)] |
| Average ICU cost (per day) | $2200 | [[34](#_ENREF_27), [35](#_ENREF_28)] |
| Antiviral cost per course | $26.2 | [[35](#_ENREF_28)] |
| Antiviral dispensing cost per course | $32.96 | [[35](#_ENREF_28)] |
| Antiviral shelf life (years) | 5 | [[56](#_ENREF_49)] |
| Mean time between pandemics (years) | 30.3 | - |
| Vaccine cost per dose | $18.93 | [[35](#_ENREF_28)] |
| Vaccine delivery cost | $10.57 | [[35](#_ENREF_28)] |
| Time and travel cost to obtain a vaccine | $16.49 | [[35](#_ENREF_28)] |
| Discount Rate (annually) | 3% | [[38](#_ENREF_31)] |

* Percentages of infections occurring in household, school/workplace, and community settings were used to derive interpersonal contact parameters.

**Table A1.2: Location specific infection rates**

| **Location** | **Pandemic transmissibility** | | |
| --- | --- | --- | --- |
|  | **R = 1.5** | **R =1.9** | **R = 2.7** |
| **Households** | 37% | 39% | 39% |
| **Hubs (schools, workplaces)** | 37% | 35% | 34% |
| **Community** | 20% | 24% | 26% |
| **Imported** | 6% | 2% | 1% |

The percentage of infections occurring in households, hubs (schools and workplaces), the general community, and those imported from outside the community are given for the no-intervention moderate and extreme pandemic scenarios.

**Figure A1.1: conceptual framework of population contact network**

**Community**

**Schools**

**Workplaces**

**Schools**

**Hub**

**Mixing group**

**Households**

**Figure A1.2: Age-specific infection rates**

The percentage of each age group that became infected (symptomatic and asymptomatic infection) is given for three pandemic scenarios: mild (R = 1.5, blue), moderate (R = 1.9, red) and extreme (R = 2.7, green).

**Additional Results**

In main paper, we reported life years saved, total costs and cost effectiveness of different vaccination strategies without reporting the productivity losses due to death (caused by the severity of the modelled pandemic scenarios) for *moderate* and *severe* pandemics. We, therefore, present additional results for a *mild* pandemic, and further present the results of total cost and cost effectiveness of those vaccination strategies including the productivity losses due to death cause for all modelled pandemics (*severe*, *moderate* and *mild*). The indirect production losses due to death were derived using a human capital method, based on the net present value of future earnings for an average age person in each age group. The additional results are shown in the following tables (Table A1.3 – Table A1.11).

**Additional results for mild pandemics without productivity losses due to death**

Table A1.3: Effectiveness (LYS per 10,000) for mild pandemics (R = 1.5 and CFR = 0.03%)

|  | **Mild pandemics (R = 1.5 and CFR = 0.03%)** | | |
| --- | --- | --- | --- |
| **Scenarios** | **SD and AV intervention** | **Life years saved (LYS) per 10,000 population** | |
|  |  | **Pre-emptive vaccination** | **Reactive vaccination** |
| **Scenario 1**: no pandemic | none | - | - |
|  | | **Pre-emptive**  **+Reactive vaccination** | **Reactive vaccination** |
| **Scenario 2**: pandemic with virus which does not match pre-emptive vaccine i.e. vaccine efficacy of 0% | none | 1* | 1* |
|  | 2 weeks SD + AV | 19 | 19 |
|  | 8 weeks SD + AV | 20 | 20 |
|  | | **Pre-emptive vaccination** | **Reactive vaccination** |
| **Scenario 3**: pandemic with virus which matches pre-emptive vaccine with efficacy of 30% | none | 21 | 1* |
|  | 2 weeks SD + AV | 23 | 19 |
|  | 8 weeks SD + AV | 23 | 20 |
|  | | **Pre-emptive vaccination** | **Reactive vaccination** |
| **Scenario 4**: pandemic with virus which matches pre-emptive vaccine with efficacy of 75% | none | 25 | 1* |
|  | 2 weeks SD + AV | 25 | 19 |
|  | 8 weeks SD + AV | 25 | 20 |

Table A1.4: Total costs for mild pandemics (R = 1.5 and CFR = 0.03%)

|  | **Mild pandemics (R = 1.5 and CFR = 0.03%)** | | |
| --- | --- | --- | --- |
| **Scenarios** | **SD and AV intervention** | **Total cost ($) per 10,000 population** | |
|  |  | **Pre-emptive vaccination** | **Reactive vaccination** |
| **Scenario 1**: no pandemic | none | $2,430,000 | $0 |
|  | | **Pre-emptive + Reactive vaccination** | **Reactive vaccination** |
| **Scenario 2**: pandemic with virus which does not match pre-emptive vaccine i.e. vaccine efficacy of 0% | none | $4,250,000 | $1,820,000 |
|  | 2 weeks SD + AV | $4,720,000 | $2,290,000 |
|  | 8 weeks SD + AV | $4,750,000 | $2,320,000 |
| Unmitigated pandemic cost (no intervention) | | $1,370,000 | |
|  | | **Pre-emptive vaccination** | **Reactive vaccination** |
| **Scenario 3**: pandemic with virus which matches pre-emptive vaccine with efficacy of 30% | none | $3,640,000 | $1,820,000 |
|  | 2 weeks SD + AV | $4,540,000 | $2,290,000 |
|  | 8 weeks SD + AV | $5,370,000 | $2,320,000 |
| Unmitigated pandemic cost (no intervention) | | $1,370,000 | |
|  | | **Pre-emptive**  **vaccination** | **Reactive vaccination** |
| **Scenario 4**: pandemic with virus which matches pre-emptive vaccine with efficacy of 75% | none | $3,620,000 | $1,820,000 |
|  | 2 weeks SD + AV | $4,320,000 | $2,290,000 |
|  | 8 weeks SD + AV | $4,660,000 | $2,320,000 |
| Unmitigated pandemic cost (no intervention) | | $1,370,000 | |

Table A1.5: Cost effectiveness for mild pandemics (R = 1.5 and CFR = 0.03%)

|  | **Mild pandemics (R = 1.5 and CFR = 0.03%)** | | |
| --- | --- | --- | --- |
| **Scenarios** | **SD and AV intervention** | **Cost ($) per LYS** | |
|  |  | **Pre-emptive vaccination** | **Reactive vaccination** |
| **Scenario 1**: no pandemic occurs | none | - | - |
|  | | **Pre-emptive + Reactive vaccination** | **Reactive vaccination** |
| **Scenario 2**: pandemic with virus which does not match pre-emptive vaccine i.e. vaccine efficacy of 0% | none | $4,250,000 | $1,820,000 |
|  | 2 weeks SD + AV | $248,421 | $120,526 |
|  | 8 weeks SD + AV | $237,500 | $116,000 |
|  | | **Pre-emptive vaccination** | **Reactive vaccination** |
| **Scenario 3**: pandemic with virus which matches pre-emptive vaccine with efficacy of 30% | none | $173,334 | $1,820,000 |
|  | 2 weeks SD + AV | $197,391 | $120,526 |
|  | 8 weeks SD + AV | $233,478 | $116,000 |
|  | | **Pre-emptive**  **Vaccination** | **Reactive vaccination** |
| **Scenario 4**: pandemic with virus which matches pre-emptive vaccine with efficacy of 75% | none | $144,800 | $1,820,000 |
|  | 2 weeks SD + AV | $172,800 | $120,526 |
|  | 8 weeks SD + AV | $186,400 | $116,000 |

**Additional results including productivity losses due to death for severe, moderate and mild pandemics**

Table A1.6: Total costs for severe pandemics (R = 2.7 and CFR = 1.5%)

|  | **Severe pandemics (R = 2.7 and CFR = 1.5%)** | | |
| --- | --- | --- | --- |
| **Scenarios** | **SD and AV intervention** | **Total cost ($) per 10,000 population** | |
|  |  | **Pre-emptive vaccination** | **Reactive vaccination** |
| **Scenario 1**: no pandemic | none | $2,430,000 | $0 |
|  | | **Pre-emptive + Reactive vaccination** | **Reactive vaccination** |
| **Scenario 2**: pandemic with virus which does not match pre-emptive vaccine i.e. vaccine efficacy of 0% | none | $76,410,000 | $73,980,000 |
|  | 8 weeks SD + AV | $53,430,000 | $51,000,000 |
|  | Sustained SD + AV | $27,520,000 | $25,090,000 |
| Unmitigated pandemic cost (no intervention) | | $73,270,000 | |
|  | | **Pre-emptive vaccination** | **Reactive vaccination** |
| **Scenario 3**: pandemic with virus which matches pre-emptive vaccine with efficacy of 30% | none | $40,630,000 | $73,980,000 |
|  | 8 weeks SD + AV | $21,830,000 | $51,000,000 |
|  | Sustained SD + AV | $15,410,000 | $25,090,000 |
| Unmitigated pandemic cost (no intervention) | | $73270000 | |
|  | | **Pre-emptive**  **Vaccination** | **Reactive vaccination** |
| **Scenario 4**: pandemic with virus which matches pre-emptive vaccine with efficacy of 75% | none | $4,400,000 | $73,980,000 |
|  | 8 weeks SD + AV | $5,620,000 | $51,000,000 |
|  | Sustained SD + AV | $7,440,000 | $25,090,000 |
| Unmitigated pandemic cost (no intervention) | | $73,270,000 | |

Table A1.7: Total costs for moderate pandemics (R = 1.9 and CFR = 0.25%)

|  | **Moderate pandemics (R = 1.9 and CFR = 0.25%)** | | |
| --- | --- | --- | --- |
| **Scenarios** | **SD and AV intervention** | **Total cost ($) per 10,000 population** | |
|  |  | **Pre-emptive vaccination** | **Reactive vaccination** |
| **Scenario 1**: no pandemic | none | $2,430,000 | $0 |
|  | | **Pre-emptive + Reactive vaccination** | **Reactive vaccination** |
| **Scenario 2**: pandemic with virus which does not match pre-emptive vaccine i.e. vaccine efficacy of 0% | none | $14,110,000 | $11,680,000 |
|  | 8 weeks SD + AV | $11,030,000 | $8,600,000 |
|  | Sustained SD + AV | $11,250,000 | $8,820,000 |
| Unmitigated pandemic cost (no intervention) | | $10,870,000 | |
|  | | **Pre-emptive vaccination** | **Reactive vaccination** |
| **Scenario 3**: pandemic with virus which matches pre-emptive vaccine with efficacy of 30% | none | $6,760,000 | $11,680,000 |
|  | 8 weeks SD + AV | $5,870,000 | $8,600,000 |
|  | Sustained SD + AV | $7,420,000 | $8,820,000 |
| Unmitigated pandemic cost (no intervention) | | $10,870,000 | |
|  | | **Pre-emptive**  **vaccination** | **Reactive vaccination** |
| **Scenario 4**: pandemic with virus which matches pre-emptive vaccine with efficacy of 75% | none | $3,700,000 | $11,680,000 |
|  | 8 weeks SD + AV | $4,880,000 | $8,600,000 |
|  | Sustained SD + AV | $6,380,000 | $8,820,000 |
| Unmitigated pandemic cost (no intervention) | | $10,870,000 | |

Table A1.8: Total costs for mild pandemics (R = 1.5 and CFR = 0.03%)

|  | **Mild pandemics (R = 1.5 and CFR = 0.03%)** | | |
| --- | --- | --- | --- |
| **Scenarios** | **SD and AV intervention** | **Total cost ($) per 10,000 population** | |
|  |  | **Pre-emptive vaccination** | **Reactive vaccination** |
| **Scenario 1**: no pandemic | none | $2,430,000 | $0 |
|  | | **Pre-emptive + Reactive vaccination** | **Reactive vaccination** |
| **Scenario 2**: pandemic with virus which does not match pre-emptive vaccine i.e. vaccine efficacy of 0% | none | $4,640,000 | $2,210,000 |
|  | 2 weeks SD + AV | $4,830,000 | $2,400,000 |
|  | 8 weeks SD + AV | $4,840,000 | $2,410,000 |
| Unmitigated pandemic cost (no intervention) | | $1,790,000 | |
|  | | **Pre-emptive vaccination** | **Reactive vaccination** |
| **Scenario 3**: pandemic with virus which matches pre-emptive vaccine with efficacy of 30% | none | $3,710,000 | $2,210,000 |
|  | 2 weeks SD + AV | $4,590,000 | $2,400,000 |
|  | 8 weeks SD + AV | $5,420,000 | $2,410,000 |
| Unmitigated pandemic cost (no intervention) | | $1,790,000 | |
|  | | **Pre-emptive**  **vaccination** | **Reactive vaccination** |
| **Scenario 4**: pandemic with virus which matches pre-emptive vaccine with efficacy of 75% | none | $3,630,000 | $2,210,000 |
|  | 2 weeks SD + AV | $4,330,000 | $2,400,000 |
|  | 8 weeks SD + AV | $4,670,000 | $2,410,000 |
| Unmitigated pandemic cost (no intervention) | | $1,790,000 | |

Table A1.9: Cost effectiveness for severe pandemics (R = 2.7 and CFR = 1.5%)

|  | **Severe pandemics (R = 2.7 and CFR = 1.5%)** | | |
| --- | --- | --- | --- |
| **Scenarios** | **SD and AV intervention** | **Cost ($) per LYS** | |
|  |  | **Pre-emptive vaccination** | **Reactive vaccination** |
| **Scenario 1**: no pandemic occurs | none | - | - |
|  | | **Pre-emptive + Reactive vaccination** | **Reactive vaccination** |
| **Scenario 2**: pandemic with virus which does not match pre-emptive vaccine i.e. vaccine efficacy of 0% | none | $8,490,000 | $8,220,000 |
|  | 8 weeks SD + AV | $43,831 | $41,838 |
|  | Sustained SD + AV | $10,118 | $9,224 |
|  | | **Pre-emptive vaccination** | **Reactive vaccination** |
| **Scenario 3**: pandemic with virus which matches pre-emptive vaccine with efficacy of 30% | none | $24,185 | $8,220,000 |
|  | 8 weeks SD + AV | $8,029 | $41,838 |
|  | Sustained SD + AV | $4,917 | $9,224 |
|  | | **Pre-emptive**  **vaccination** | **Reactive vaccination** |
| **Scenario 4**: pandemic with virus which matches pre-emptive vaccine with efficacy of 75% | none | $1,258 | $8,220,000 |
|  | 8 weeks SD + AV | $1,603 | $41,838 |
|  | Sustained SD + AV | $2,120 | $9,224 |

Table A1.10: Cost effectiveness for moderate pandemics (R = 1.9 and CFR = 0.25%)

|  | **Moderate pandemics (R = 1.9 and CFR = 0.25%)** | | |
| --- | --- | --- | --- |
| **Scenarios** | **SD and AV intervention** | **Cost ($) per LYS** | |
|  |  | **Pre-emptive vaccination** | **Reactive vaccination** |
| **Scenario 1**: no pandemic occurs | none | - | - |
|  | | **Pre-emptive + Reactive vaccination** | **Reactive vaccination** |
| **Scenario 2**: pandemic with virus which does not match pre-emptive vaccine i.e. vaccine efficacy of 0% | none | $3,527,500 | $2,920,000 |
|  | 8 weeks SD + AV | $43,597 | $33,992 |
|  | Sustained SD + AV | $26,722 | $20,950 |
|  | | **Pre-emptive vaccination** | **Reactive vaccination** |
| **Scenario 3**: pandemic with virus which matches pre-emptive vaccine with efficacy of 30% | none | $21,667 | $2,920,000 |
|  | 8 weeks SD + AV | $14,145 | $33,992 |
|  | Sustained SD + AV | $17,296 | $20,950 |
|  | | **Pre-emptive**  **vaccination** | **Reactive vaccination** |
| **Scenario 4**: pandemic with virus which matches pre-emptive vaccine with efficacy of 75% | none | $8,168 | $2,920,000 |
|  | 8 weeks SD + AV | $10,609 | $33,992 |
|  | Sustained SD + AV | $13,810 | $20,950 |

Table A1.11: Cost effectiveness for mild pandemics (R = 1.5 and CFR = 0.03%)

|  | **Mild pandemics (R = 1.5 and CFR = 0.03%)** | | |
| --- | --- | --- | --- |
| **Scenarios** | **SD and AV intervention** | **Cost ($) per LYS** | |
|  |  | **Pre-emptive vaccination** | **Reactive vaccination** |
| **Scenario 1**: no pandemic occurs | none | - | - |
|  | | **Pre-emptive + Reactive vaccination** | **Reactive vaccination** |
| **Scenario 2**: pandemic with virus which does not match pre-emptive vaccine i.e. vaccine efficacy of 0% | none | $4,640,000 | $2,210,000 |
|  | 2 weeks SD + AV | $254,211 | $126,316 |
|  | 8 weeks SD + AV | $242,000 | $120,500 |
|  | | **Pre-emptive vaccination** | **Reactive vaccination** |
| **Scenario 3**: pandemic with virus which matches pre-emptive vaccine with efficacy of 30% | none | $176,667 | $2,210,000 |
|  | 2 weeks SD + AV | $199,565 | $126,316 |
|  | 8 weeks SD + AV | $235,652 | $120,500 |
|  | | **Pre-emptive**  **vaccination** | **Reactive vaccination** |
| **Scenario 4**: pandemic with virus which matches pre-emptive vaccine with efficacy of 75% | none | $145,200 | $2,210,000 |
|  | 2 weeks SD + AV | $173,200 | $126,316 |
|  | 8 weeks SD + AV | $186,800 | $120,500 |

In main result section we presented average cost effectiveness ratio as cost/LYS. An estimation of confidence interval (95% confidence) for the cost effectiveness ratio for moderate and severe pandemics for scenario 2 is presented in Figure A1.3.

**Figure A1.3: An estimation of confidence interval for cost effectiveness ratio**


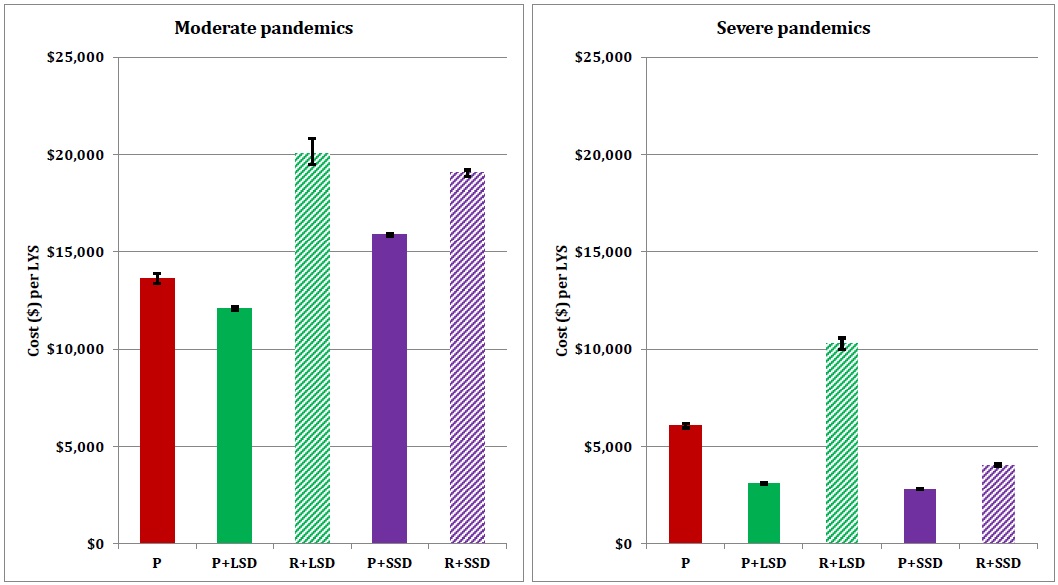


Intervention strategies are labelled as **R**: *Reactive* vaccination, **P**: *Pre-emptive* vaccination, **R+LSD**: *Reactive* vaccination + Limited Social Distancing (8 weeks of SC+CCR) + AV, **P+LSD**: *Pre-emptive* vaccination + Limited Social Distancing + AV, **R+SSD**: *Reactive* vaccination + Sustained Social Distancing (Sustained SC+CCR) + AV, **P+SSD**: *Pre-emptive* vaccination + Sustained Social Distancing + AV, SC – School Closure, CCR – Community Contact Reduction, AV – Antiviral treatment for cases and prophylaxis for their household members, LSD – Limited Social Distancing, SSD – Sustained Social Distancing. All LSD and SSD interventions integrate the use of antivirals (AV). Vaccine efficacy of 30% and 75% is assumed for pre-emptive and reactive vaccines respectively; 100% coverage is assumed.

**Additional Sensitivity Analyses**

In main manuscript, we performed sensitivity analysis of key model parameters such as pre-emptive vaccination coverage, the time between pre-pandemic vaccine renewals, the expected time between pandemics and the chance of pre-pandemic vaccine mismatch. Further sensitivity analyses for variation in weekly wages and inclusion/exclusion of productivity losses due to illness are presented in Figure A1.4 and Figure A1.5 respectively.

**Figure A1.4: Impact of different weekly wages on cost effectiveness ratio**

**
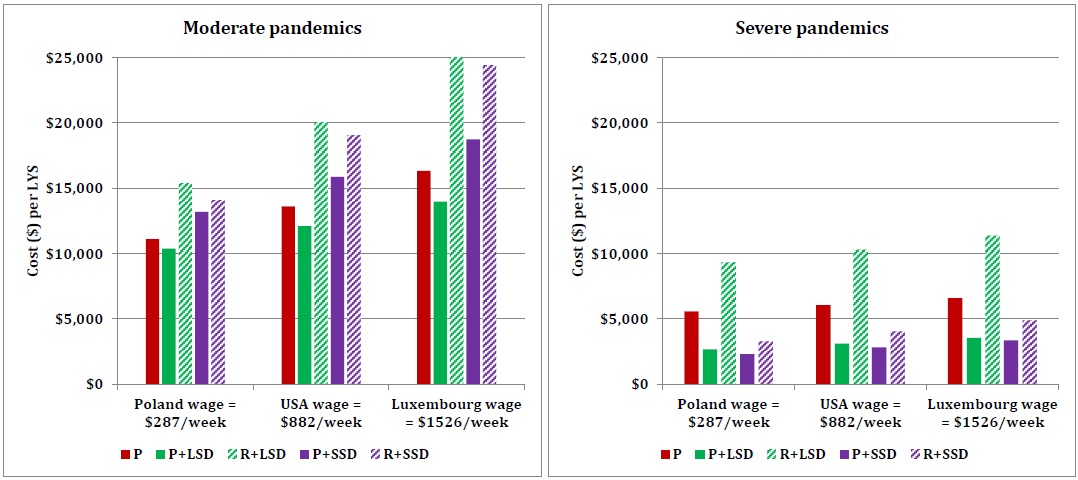
**

Intervention strategies are labelled as **R**: *Reactive* vaccination, **P**: *Pre-emptive* vaccination, **R+LSD**: *Reactive* vaccination + Limited Social Distancing (8 weeks of SC+CCR) + AV, **P+LSD**: *Pre-emptive* vaccination + Limited Social Distancing + AV, **R+SSD**: *Reactive* vaccination + Sustained Social Distancing (Sustained SC+CCR) + AV, **P+SSD**: *Pre-emptive* vaccination + Sustained Social Distancing + AV, SC – School Closure, CCR – Community Contact Reduction, AV – Antiviral treatment for cases and prophylaxis for their household members, LSD – Limited Social Distancing, SSD – Sustained Social Distancing. All LSD and SSD interventions integrate the use of antivirals (AV). Vaccine efficacy of 30% and 75% is assumed for pre-emptive and reactive vaccines respectively; 100% coverage is assumed.

**Figure A1.5: Impact of inclusion and exclusion of productivity losses due to illness**


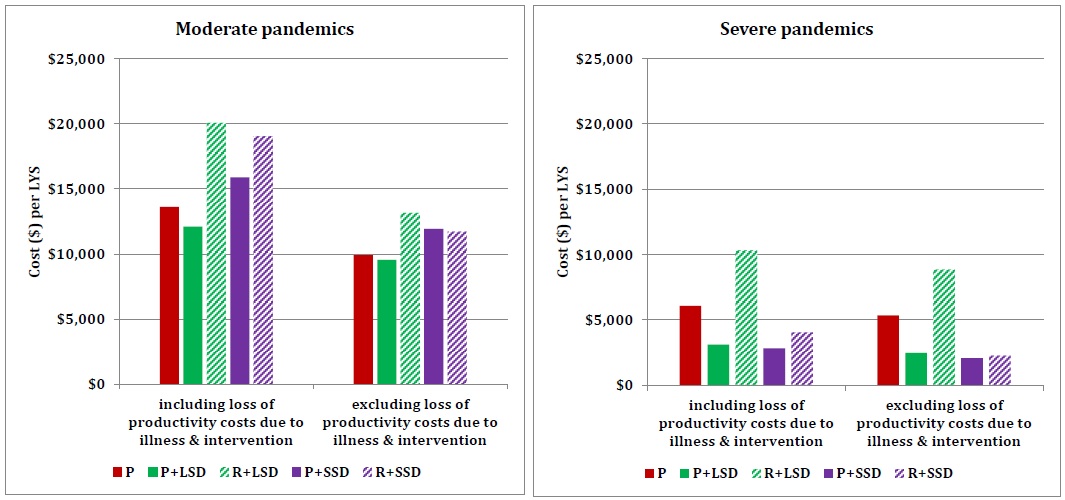


Intervention strategies are labelled as **R**: *Reactive* vaccination, **P**: *Pre-emptive* vaccination, **R+LSD**: *Reactive* vaccination + Limited Social Distancing (8 weeks of SC+CCR) + AV, **P+LSD**: *Pre-emptive* vaccination + Limited Social Distancing + AV, **R+SSD**: *Reactive* vaccination + Sustained Social Distancing (Sustained SC+CCR) + AV, **P+SSD**: *Pre-emptive* vaccination + Sustained Social Distancing + AV, SC – School Closure, CCR – Community Contact Reduction, AV – Antiviral treatment for cases and prophylaxis for their household members, LSD – Limited Social Distancing, SSD – Sustained Social Distancing. All LSD and SSD interventions integrate the use of antivirals (AV). Vaccine efficacy of 30% and 75% is assumed for pre-emptive and reactive vaccines respectively; 100% coverage is assumed.

## References

1. **Australian Burea of Statistics: Census Data Online** [<http://abs.gov.au/websitedbs/D3310114.nsf/home/Census+data>]

2. Papoulis A: *Probability, random variables and stochastic processes.* 2nd edn. New York: McGraw-Hill; 1984.

3. Monto A, Koopman J, Longini IMJ: **Tecumseh study of illness XIII. Influenza infection and disease, 1976-1981.** *Am J Epidemiol* 1985, **121:**811-822.

4. Milne GJ, Kelso JK, Kelly HA, Huband ST, McVernon J: **A small community model for the transmission of infectious diseases: comparison of school closure as an intervention in individual-based models of an influenza pandemic.** *PLoS ONE* 2008, **3:**e4005.

5. Carrat F, Vergu E, Ferguson NM, Lemaitre M, Cauchemez S, Leach S, Valleron A-J: **Time lines of infection and disease in human influenza: a review of volunteer challenge studies.** *Am J Epidemiol* 2008, **167:**775-785.

6. Fraser C, Christl DA, Cauchemez S, Hanage WP, Van Kerkhove MD, Hollingsworth TD, Griffin J, Baggaley RF, Jenkins HE, Lyons EJ, et al: **Pandemic potential of a strain of influenza A (H1N1): early findings.** *Science* 2009, **324:**1557-1561.

7. Fox JP, Hall CE, Cooney MK, Foy HM: **Influenzavirus infections in Seattle families, 1975-1979.** *Am J Epidemiol* 1982, **116:**212-227.

8. Ferguson NM, Cummings DAT, Fraser C, Cajka JC, Cooley PC, Burke DS: **Strategies for mitigating an influenza pandemic.** *Nature* 2006, **442:**448-452.

9. Longini IM, Jr., Nizam A, Xu S, Ungchusak K, Hanshaoworakul W, Cummings DAT, Halloran ME: **Containing pandemic influenza at the source.** *Science* 2005, **309:**1083-1088.

10. Yang Y, Longini IM, Jr., Halloran ME: **Design and evaluation of prophylactic interventions using infectious disease incidence data from close contact groups.** *Appl Statist* 2006, **55:**317-330.

11. Germann TC, Kadau K, Longini IM, Jr., Macken CA: **Mitigation strategies for pandemic influenza in the United States.** *PNAS* 2006, **103:**5935-5941.

12. Halloran ME, Ferguson NM, Eubank S, Longini IM, Cummings DAT, Lewis B, Xu S, Fraser C, Vullikanti A, Germann TC: **Modeling targeted layered containment of an influenza pandemic in the United States.** *PNAS* 2008, **105:**4639.

13. Longini I, Halloran M, Nizam A, Yang Y: **Containing pandemic influenza with antiviral agents.** *Am J Epidemiol* 2004, **159:**623-633.

14. Hayden FG, Belshe R, Villanueva C, Lanno R, Hughes C, Small I, Dutkowski R, Ward P, Carr J: **Management of influenza in households: a prospective, randomized comparison of oseltamivir treatment with or without postexposure prophylaxis.** *The Journal of Infectious Diseases* 2004, **189:**440-449.

15. Moscona A: **Neuraminidase inhibitors for influenza.** *N Engl J Med* 2005, **353:**1363-1373.

16. [Plosker GL](http://www.ncbi.nlm.nih.gov/pubmed?term=Plosker%20GL%5BAuthor%5D&cauthor=true&cauthor_uid=22788239): **A/H5N1 prepandemic influenza vaccine (whole virion, vero cell-derived, inactivated)**, Drugs 2012, 72(11):1543-57.

17. [van der Velden MV](http://www.ncbi.nlm.nih.gov/pubmed?term=van%20der%20Velden%20MV%5BAuthor%5D&cauthor=true&cauthor_uid=24041789), [Fritz R](http://www.ncbi.nlm.nih.gov/pubmed?term=Fritz%20R%5BAuthor%5D&cauthor=true&cauthor_uid=24041789), [Pöllabauer EM](http://www.ncbi.nlm.nih.gov/pubmed?term=P%C3%B6llabauer%20EM%5BAuthor%5D&cauthor=true&cauthor_uid=24041789), [Portsmouth D](http://www.ncbi.nlm.nih.gov/pubmed?term=Portsmouth%20D%5BAuthor%5D&cauthor=true&cauthor_uid=24041789), [Howard MK](http://www.ncbi.nlm.nih.gov/pubmed?term=Howard%20MK%5BAuthor%5D&cauthor=true&cauthor_uid=24041789), [Kreil TR](http://www.ncbi.nlm.nih.gov/pubmed?term=Kreil%20TR%5BAuthor%5D&cauthor=true&cauthor_uid=24041789), [Dvorak T](http://www.ncbi.nlm.nih.gov/pubmed?term=Dvorak%20T%5BAuthor%5D&cauthor=true&cauthor_uid=24041789), [Fritsch S](http://www.ncbi.nlm.nih.gov/pubmed?term=Fritsch%20S%5BAuthor%5D&cauthor=true&cauthor_uid=24041789), [Vesikari T](http://www.ncbi.nlm.nih.gov/pubmed?term=Vesikari%20T%5BAuthor%5D&cauthor=true&cauthor_uid=24041789), [Diez-Domingo J](http://www.ncbi.nlm.nih.gov/pubmed?term=Diez-Domingo%20J%5BAuthor%5D&cauthor=true&cauthor_uid=24041789), [Richmond P](http://www.ncbi.nlm.nih.gov/pubmed?term=Richmond%20P%5BAuthor%5D&cauthor=true&cauthor_uid=24041789), [Lee BW](http://www.ncbi.nlm.nih.gov/pubmed?term=Lee%20BW%5BAuthor%5D&cauthor=true&cauthor_uid=24041789), [Kistner O](http://www.ncbi.nlm.nih.gov/pubmed?term=Kistner%20O%5BAuthor%5D&cauthor=true&cauthor_uid=24041789), [Ehrlich HJ](http://www.ncbi.nlm.nih.gov/pubmed?term=Ehrlich%20HJ%5BAuthor%5D&cauthor=true&cauthor_uid=24041789), [Barrett PN](http://www.ncbi.nlm.nih.gov/pubmed?term=Barrett%20PN%5BAuthor%5D&cauthor=true&cauthor_uid=24041789), [Aichinger G](http://www.ncbi.nlm.nih.gov/pubmed?term=Aichinger%20G%5BAuthor%5D&cauthor=true&cauthor_uid=24041789): **Safety and Immunogenicity of a Vero Cell Culture-Derived Whole-Virus H5N1 Influenza Vaccine in a Pediatric Population**, J Infect Dis 2013.

18. [Herbinger KH](http://www.ncbi.nlm.nih.gov/pubmed?term=Herbinger%20KH%5BAuthor%5D&cauthor=true&cauthor_uid=24047817), [von Sonnenburg F](http://www.ncbi.nlm.nih.gov/pubmed?term=von%20Sonnenburg%20F%5BAuthor%5D&cauthor=true&cauthor_uid=24047817), [Nothdurft HD](http://www.ncbi.nlm.nih.gov/pubmed?term=Nothdurft%20HD%5BAuthor%5D&cauthor=true&cauthor_uid=24047817), [Perona P](http://www.ncbi.nlm.nih.gov/pubmed?term=Perona%20P%5BAuthor%5D&cauthor=true&cauthor_uid=24047817), [Borkowski A](http://www.ncbi.nlm.nih.gov/pubmed?term=Borkowski%20A%5BAuthor%5D&cauthor=true&cauthor_uid=24047817), [Fragapane E](http://www.ncbi.nlm.nih.gov/pubmed?term=Fragapane%20E%5BAuthor%5D&cauthor=true&cauthor_uid=24047817), [Nicolay U](http://www.ncbi.nlm.nih.gov/pubmed?term=Nicolay%20U%5BAuthor%5D&cauthor=true&cauthor_uid=24047817), [Clemens R](http://www.ncbi.nlm.nih.gov/pubmed?term=Clemens%20R%5BAuthor%5D&cauthor=true&cauthor_uid=24047817)**: A phase II study of an investigational tetravalent influenza vaccine formulation combining MF59®: Adjuvanted, pre-pandemic, A/H5N1 vaccine and trivalent seasonal influenza vaccine in healthy adults.** Hum Vaccin Immunother 2013, 10(1).

19. [Czajka H](http://www.ncbi.nlm.nih.gov/pubmed?term=Czajka%20H%5BAuthor%5D&cauthor=true&cauthor_uid=23362618), [Unal S](http://www.ncbi.nlm.nih.gov/pubmed?term=Unal%20S%5BAuthor%5D&cauthor=true&cauthor_uid=23362618), [Ulusoy S](http://www.ncbi.nlm.nih.gov/pubmed?term=Ulusoy%20S%5BAuthor%5D&cauthor=true&cauthor_uid=23362618), [Usluer G](http://www.ncbi.nlm.nih.gov/pubmed?term=Usluer%20G%5BAuthor%5D&cauthor=true&cauthor_uid=23362618), [Strus A](http://www.ncbi.nlm.nih.gov/pubmed?term=Strus%20A%5BAuthor%5D&cauthor=true&cauthor_uid=23362618), [Sennaroglu E](http://www.ncbi.nlm.nih.gov/pubmed?term=Sennaroglu%20E%5BAuthor%5D&cauthor=true&cauthor_uid=23362618), [Guzik J](http://www.ncbi.nlm.nih.gov/pubmed?term=Guzik%20J%5BAuthor%5D&cauthor=true&cauthor_uid=23362618), [Topeli Iskit A](http://www.ncbi.nlm.nih.gov/pubmed?term=Topeli%20Iskit%20A%5BAuthor%5D&cauthor=true&cauthor_uid=23362618), [Dargiewicz A](http://www.ncbi.nlm.nih.gov/pubmed?term=Dargiewicz%20A%5BAuthor%5D&cauthor=true&cauthor_uid=23362618), [Musial D](http://www.ncbi.nlm.nih.gov/pubmed?term=Musial%20D%5BAuthor%5D&cauthor=true&cauthor_uid=23362618), [Caylan R](http://www.ncbi.nlm.nih.gov/pubmed?term=Caylan%20R%5BAuthor%5D&cauthor=true&cauthor_uid=23362618), [Dziduch J](http://www.ncbi.nlm.nih.gov/pubmed?term=Dziduch%20J%5BAuthor%5D&cauthor=true&cauthor_uid=23362618), [Eskioglu E](http://www.ncbi.nlm.nih.gov/pubmed?term=Eskioglu%20E%5BAuthor%5D&cauthor=true&cauthor_uid=23362618), [Hasiec B](http://www.ncbi.nlm.nih.gov/pubmed?term=Hasiec%20B%5BAuthor%5D&cauthor=true&cauthor_uid=23362618),[Cwinarowiczliwa E](http://www.ncbi.nlm.nih.gov/pubmed?term=Cwinarowiczliwa%20E%5BAuthor%5D&cauthor=true&cauthor_uid=23362618), [Belli R](http://www.ncbi.nlm.nih.gov/pubmed?term=Belli%20R%5BAuthor%5D&cauthor=true&cauthor_uid=23362618), [Abdel-Messih IA](http://www.ncbi.nlm.nih.gov/pubmed?term=Abdel-Messih%20IA%5BAuthor%5D&cauthor=true&cauthor_uid=23362618), [Beygo J](http://www.ncbi.nlm.nih.gov/pubmed?term=Beygo%20J%5BAuthor%5D&cauthor=true&cauthor_uid=23362618), [Fragapane E](http://www.ncbi.nlm.nih.gov/pubmed?term=Fragapane%20E%5BAuthor%5D&cauthor=true&cauthor_uid=23362618): **A phase II, randomised clinical trial to demonstrate the non-inferiority of low-dose MF59-adjuvanted pre-pandemicA/H5N1 influenza vaccine in adult and elderly subjects**. J Prev Med Hyg 2012, 53(3):136-42.

20. [Vesikari T](http://www.ncbi.nlm.nih.gov/pubmed?term=Vesikari%20T%5BAuthor%5D&cauthor=true&cauthor_uid=22192847), [Forstén A](http://www.ncbi.nlm.nih.gov/pubmed?term=Forst%C3%A9n%20A%5BAuthor%5D&cauthor=true&cauthor_uid=22192847), [Herbinger KH](http://www.ncbi.nlm.nih.gov/pubmed?term=Herbinger%20KH%5BAuthor%5D&cauthor=true&cauthor_uid=22192847), [Cioppa GD](http://www.ncbi.nlm.nih.gov/pubmed?term=Cioppa%20GD%5BAuthor%5D&cauthor=true&cauthor_uid=22192847), [Beygo J](http://www.ncbi.nlm.nih.gov/pubmed?term=Beygo%20J%5BAuthor%5D&cauthor=true&cauthor_uid=22192847), [Borkowski A](http://www.ncbi.nlm.nih.gov/pubmed?term=Borkowski%20A%5BAuthor%5D&cauthor=true&cauthor_uid=22192847), [Groth N](http://www.ncbi.nlm.nih.gov/pubmed?term=Groth%20N%5BAuthor%5D&cauthor=true&cauthor_uid=22192847), [Bennati M](http://www.ncbi.nlm.nih.gov/pubmed?term=Bennati%20M%5BAuthor%5D&cauthor=true&cauthor_uid=22192847), [von Sonnenburg F](http://www.ncbi.nlm.nih.gov/pubmed?term=von%20Sonnenburg%20F%5BAuthor%5D&cauthor=true&cauthor_uid=22192847): **Safety and immunogenicity of an MF59(®)-adjuvanted A/H5N1 pre-pandemic influenza vaccine in adults and the elderly**. [Vaccine](http://www.ncbi.nlm.nih.gov/pubmed/22192847) 2012, 30(7):1388-96.

21. Leroux-Roels I, Borkowski A, Vanwolleghem T, Dramé M, Clement F, Hons E, Devaster J-M, Leroux-Roels G: **Antigen sparing and cross-reactive immunity with an adjuvanted rH5N1 prototype pandemic infl uenza vaccine: a randomised controlled trial**. Lancet 2007, 370:580–589.

22. Leroux-Roels I, Bernhard R, Gérard P, Dramé M, Hanon E, Leroux-Roels G: **Broad clade 2 cross-reactive immunity induced by an adjuvanted clade 1 rH5N1 pandemic influenza vaccine**. PLoS One 2008, 3(2):e1665.

23. Govaert TM, Sprenger MJ, Dinant GJ, Aretz K, Masurel N, Knottnerus JA: **Immune response to influenza vaccination of elderly people: A randomized double-blind placebo-controlled trial**. Vaccine 1994, 12(13):1185-9.

24. Milne G, Kelso J, Kelly H: **Strategies for mitigating an influenza pandemic with pre-pandemic H5N1 vaccines**. J R Soc Interface 2010, 7:573–586.

25. Greenberg ME, Lai MH, Hartel GF, Wichems CH, Gittleson C, Bennet J, Dawson G, Hu W, Leggio C, Washington D: **Response to a monovalent 2009 influenza A (H1N1) vaccine**. N Engl J Med 2009, 361(25):2405–2413.

26. Mylius SD, Hagenaars TJ, Lugner AK, Wallinga J: **Optimal allocation of pandemic influenza vaccine depends on age, risk and timing**. Vaccine 2008, 26:3742–3749.

27. Hayden FG, Howard WA, Palkonyay L, Kieny MP: **Report of the 5th meeting on the evaluation of pandemic influenza prototype vaccines in clinical trials: World Health Organization, Geneva, Switzerland, 12-13 February 2009.** *Vaccine* 2009, **27:**4079-4089.

28. Halder N, Kelso J, Milne G: **Developing guidelines for school closure interventions to be used during a future influenza pandemic.** *BMC Infect Dis* 2010, **10:**221.

29. Halder N, Kelso J, Milne G: **Analysis of the effectiveness of interventions used during the 2009 H1N1 influenza pandemic.** *BMC Public Health* 2010, **10:**168.

30. Kelso JK, Halder N, Milne GJ: **The impact of case diagnosis coverage and diagnosis delays on the effectiveness of antiviral strategies in mitigating pandemic influenza A/H1N1 2009.** *PLoS ONE* 2010, **5:**e13797.

31. Lee VJ, Phua KH, Chen MI, Chow A, Ma S, Goh KT, Leo YS: **Economics of neuraminidase inhibitor stockpiling for pandemic influenza, Singapore.** *Emerg Infect Dis* 2006, **12:**95-102.

32. Lee VJ, Tok MY, Chow VT, Phua KH, Ooi EE, Tambyah PA, Chen MI: **Economic analysis of pandemic influenza vaccination strategies in Singapore.** *PLoS ONE* 2009, **4:**e7108.

33. Baguelin M, Hoek AJV, Jit M, Flasche S, White PJ, Edmunds WJ: **Vaccination against pandemic influenza A/H1N1v in England: a real-time economic evaluation.** *Vaccine* 2010, **28:**2370-2384.

34. Keren R, Zaoutis TE, Saddlemire S, Luan XQ, Coffin SE: **Direct medical cost of influenza-related hospitalizations in children.** *Pediatrics* 2006, **118:**e1321-e1327.

35. Sander B, Nizam A, Garrison LP, Jr., Postma MJ, Halloran EM, Longini IM, Jr.: **Economic evaluation of influenza pandemic mitigation strategies in the United States using a stochastic microsimulation transmission model.** *Value in Health* 2009, **12:**226-233.

36. Perlroth DJ, Glass RJ, Davey VJ, Cannon D, Garber AM, Owens DK: **Health outcomes and costs of community mitigation strategies for an influenza pandemic in the United States.** *Clin Infect Dis* 2009, **50:**165-174.

37. **U.S. Bureau of Labor Statistics** [<http://www.bls.gov>]

38. World Health Organization: **Making choices in health: WHO guide to cost-effectiveness analysis.** In *Book Making choices in health: WHO guide to cost-effectiveness analysis* (Editor ed.^eds.): World Health Organization; 2003.

39. **USA Historical Consumer Price Index** [<http://rateinflation.com/consumer-price-index/usa-historical-cpi.php>]

40. Kelly H, Mercer G, Fielding J, Dowse G, Glass K, Carcione D, Grant KA, Effler PV, Lester RA, Gravenor MB: **Pandemic (H1N1) 2009 influenza community transmission was established in one Australian state when the virus was first identified in North America.** *PLoS ONE* 2010, **5:**e11341.

41. Nishiura H, Chowell G, Safan M, Castillo-Chavez C: **Pros and cons of estimating the reproduction number from early epidemic growth rate of influenza A(H1N1) 2009.** *Theoretical Biology and Medical Modelling* 2010, **7**.

42. Dawood FS, Iuliano AD, Reed C, Meltzer MI, Shay DK, Cheng PY, Bandaranayake D, Breiman RF, Brooks WA, Buchy P: **Estimated global mortality associated with the first 12 months of 2009 pandemic influenza A H1N1 virus circulation: a modelling study.** *The Lancet Infectious Diseases* 2012.

43. Vynnycky E, Edmunds W: **Analyses of the 1957 (Asian) influenza pandemic in the United Kingdom and the impact of school closures.** *Epidemiol Infect* 2008, **136:**166-179.

44. Viboud C, Tam T, Fleming D, Handel A, Miller MA, Simonsen L: **Transmissibility and mortality impact of epidemic and pandemic influenza, with emphasis on the unusually deadly 1951 epidemic.** *Vaccine* 2006, **24:**6701-6707.

45. Simonsen L, Clarke MJ, Schonberger LB, Arden NH, Cox NJ, Fukuda K: **Pandemic versus epidemic influenza mortality: a pattern of changing age distribution.** *J Infect Dis* 1998, **178:**53-60.

46. Glezen WP: **Emerging infections: pandemic influenza.** *Epidemiol Rev* 1996, **18:**64-76.

47. Gani R, Hughes H, Fleming D, Griffin T, Medlock J, Leach S: **Potential impact of antiviral drug use during influenza pandemic.** *Emerg Infect Dis* 2005, **11:**1355-1362.

48. Mills CE, Robins JM, Lipsitch M: **Transmissibility of 1918 pandemic influenza.** *Nature* 2004, **432:**904-906.

49. Mathews JD, McCaw CT, McBryde JMES, McCaw JM: **A Biological Model for Influenza Transmission: Pandemic Planning Implications of Asymptomatic Infection and Immunity.** *PLoS ONE* 2007**:**1-6.

50. Frost W: **Statistics of influenza morbidity with special reference to certain factors in case incidence and case fatality.** *Public Heath Report* 1920, **35:**584-597.

51. ANZIC Influenza Investigators: **Critical Care Services and 2009 H1N1 influenza in Australia and New Zealand.** *N Engl J Med* 2009, **361:**1925-1934.

52. Newall AT, Wood JG, Oudin N, MacIntyre CR: **Cost-effectiveness of pharmaceutical-based pandemic influenza mitigation strategies.** *Emerg Infect Dis* 2010, **16:**224-230.

53. Song JY, Cheong HJ, Seo YB, Kim IS, Noh JY, Heo JY, Choi WS, Lee J, Kim WJ: **Comparison of the Long-Term Immunogenicity of Two Pandemic Influenza A/H1N1 2009 Vaccines, the MF59-Adjuvanted and Unadjuvanted Vaccines, in Adults.** *Clinical and Vaccine Immunology* 2012, **19:**638-641.

54. Saitoh A, Nagai A, Tenjinbaru K, Li P, Roman F, Kato T: **Safety and persistence of immunological response 6 months after intramuscular vaccination with an AS03-adjuvanted H1N1 2009 influenza vaccine: An open-label, randomized trial in Japanese children aged 6 months to 17 years.** *Human Vaccines & Immunotherapeutics* 2012, **8:**10-19.

55. Lopez-Macaas C, Ferat-Osorio E, Tenorio-Calvo A, Isibasi A, Talavera J, Arteaga-Ruiz O, Arriaga-Pizano L, Hickman SP, Allende M, Lenhard K: **Safety and immunogenicity of a virus-like particle pandemic influenza A (H1N1) 2009 vaccine in a blinded, randomized, placebo-controlled trial of adults in Mexico.** *Vaccine* 2011, **29:**7826-7834.

56. Reddy D: **Responding to pandemic (H1N1) 2009 influenza: the role of oseltamivir.** *J Antimicrob Chemother* 2010, **65 Suppl 2:**ii35-40.

57. Cauchemez S, Carrat F, Viboud C, Valleron A, Boelle P: **A Bayesian MCMC approach to study transmission of influenza: application to household longitudinal data**. *Stat Med* 2004, **23**(22):3469-3487.

58. Cauchemez S, Valleron A-J, Boe¨lle P-Y, Flahault A, Ferguson NM: **Estimating the impact of school closure on influenza transmission from Sentinel data**. *Nature Letters* 2008, **452**:750-755.58.

59. [Kelso JK](http://www.ncbi.nlm.nih.gov/pubmed?term=Kelso%20JK%5BAuthor%5D&cauthor=true&cauthor_uid=23398722), [Halder N](http://www.ncbi.nlm.nih.gov/pubmed?term=Halder%20N%5BAuthor%5D&cauthor=true&cauthor_uid=23398722), [Milne GJ](http://www.ncbi.nlm.nih.gov/pubmed?term=Milne%20GJ%5BAuthor%5D&cauthor=true&cauthor_uid=23398722): **Vaccination strategies for future influenza pandemics: a severity-based cost effectiveness analysis**. [*BMC Infect Dis*.](http://www.ncbi.nlm.nih.gov/pubmed/?term=Vaccination+strategies+for+future+influenza+pandemics%3A+a+severity-based+cost+effectiveness+analysis) 2013 Feb 11;13:81.

60. Goodwin K, Viboud C, Simonsen L: **Antibody response to influenza vaccination in the elderly: a quantitative review**. Vaccine 2006, 24(8):1159-69.
